# Effects of calcium level and source, formic acid, and phytase on phytate degradation and the microbiota in the digestive tract of broiler chickens

**DOI:** 10.1186/s42523-021-00083-7

**Published:** 2021-03-15

**Authors:** Jochen Krieg, Daniel Borda-Molina, Wolfgang Siegert, Vera Sommerfeld, Yung Ping Chi, Hamid Reza Taheri, Dieter Feuerstein, Amélia Camarinha-Silva, Markus Rodehutscord

**Affiliations:** 1 Institute of Animal Science, University of Hohenheim, 70599 Stuttgart, Germany; 2 Department of Animal Science, Faculty of Agriculture, University of Zanjan, Zanjan, 45371-38791 Iran; 3 BASF SE, 68623 Lampertheim, Germany

**Keywords:** Calcium, Phytate, Microbiota, Functionality, Broiler chickens

## Abstract

**Background:**

Diet acidification, dietary calcium (**Ca**) level, and phytase supplementation are known influences on the microbial community in the digestive tract and on phosphorus (**P**) utilization of broiler chickens. Effects of dietary factors and microbiota on P utilization may be linked because microorganisms produce enzymes that release P from phytate (**InsP**_**6**_), the main source of P in plant feedstuffs. This study aimed to detect linkages between microbiota and InsP_6_ degradation by acidifying diets (i.e., replacing Ca carbonate (**CaCO**_**3**_) by Ca formate or adding formic acid to CaCO_3_-containing diets), varying Ca levels, and supplementing phytase in a three-factorial design. We investigated i) the microbial community and pH in the digestive tract, ii) prececal (**pc**) P and Ca digestibility, and iii) InsP_6_ degradation.

**Results:**

All factors under investigation influenced digesta pH and the microbiota composition. Predicted functionality and relative abundance of microorganisms indicated that diets influenced the potential contribution of the microbiota on InsP degradation. Values of InsP_6_ degradation and relative abundance of the strains *Lactobacillus johnsonii* and *Lactobacillus reuteri* were correlated. Phytase supplementation increased pc InsP_6_ disappearance, with differences between Ca levels, and influenced concentrations of lower inositol phosphate isomers in the digestive tract. Formic acid supplementation increased pc InsP_6_ degradation to *myo*-inositol. Replacing CaCO_3_ by Ca-formate and the high level of these Ca sources reduced pc InsP_6_ disappearance, except when the combination of CaCO_3_ + formic acid was used. Supplementing phytase to CaCO_3_ + formic acid led to the highest InsP_6_ disappearance (52%) in the crop and increased *myo*-inositol concentration in the ileum digesta. Supplementing phytase leveled the effect of high Ca content on pc InsP_6_ disappearance.

**Conclusions:**

The results point towards a contribution of changing microbial community on InsP_6_ degradation in the crop and up to the terminal ileum. This is indicated by relationships between InsP_6_ degradation and relative abundance of phosphatase-producing strains. Functional predictions supported influences of microbiota on InsP_6_ degradation. The extent of such effects remains to be clarified. InsP_6_ degradation may also be influenced by variation of pH caused by dietary concentration and solubility of the Ca in the feed.

**Supplementary Information:**

The online version contains supplementary material available at 10.1186/s42523-021-00083-7.

## Background

High utilization of phosphorus (**P**) provided by plant feedstuffs is advantageous because less or no mineral P is needed to fulfill the P requirement of animals. Plant-P is mainly bound in phytic acid [*myo*-inositol 1,2,3,4,5,6-hexakis (dihydrogen phosphate); **InsP**_**6**_] and present as phytate, which requires hydrolyzing enzymes to make P available for the animal. Hence, phytases are widely used as feed additives in non-ruminant nutrition. The main effect of phytases is the cleavage of P from InsP_6_ to increase P utilization by animals. When diets are fed with the supplementation of mineral P and calcium (**Ca**), but without supplemented phytase, prececal (**pc**) InsP_6_ degradation is reduced in broiler chickens [1]. The reduced degradation of InsP_6_ upon Ca supplementation is usually explained, among other reasons, by the formation of Ca-InsP_6_ complexes in the digestive tract at high Ca concentrations [2, 3].

It was shown that bone mineralization of broiler chickens can differ as a result of varying Ca sources when supplied in equal Ca concentrations [4] and that using different Ca sources can have other consequences, such as impacts on the microbial community of the digestive tract of broiler chickens [5]. Studies investigating the impact of dietary Ca on P utilization usually used limestone as a Ca source, which mainly consists of calcium carbonate (**CaCO**_**3**_), a compound with a high buffer and acid-binding capacity. Increasing pH in the digestive tract supports formation of Ca-InsP_6_ complexes and, thus, reduces pc InsP_6_ degradation [2, 6, 7]. Reducing intestinal pH might counteract effects of high dietary Ca concentrations. Ca salts of organic acids like Ca-formate are known to have a lower buffer capacity than CaCO_3_ [8]. Hence, replacing CaCO_3_ by Ca-formate might compensate the pH increase caused by CaCO_3_ supplementation and thereby increase InsP_6_ degradation and, as a consequence of changing the Ca source, affect the microbial community. Solubility of dietary Ca might also influence P utilization through Ca-InsP_6_ complexes or other mechanisms. Complexation of Ca and InsP_6_ was shown to be increased at pH 5 and higher [9]. Using Ca sources with higher solubility than that of CaCO_3_ might allow for a higher Ca absorption in the proximal section of the small intestine and thus reduce the amount of Ca cations available for Ca-InsP_6_ complex formation.

A decrease in pH could also be achieved by adding formic acid to diets. As reviewed by Kim et al. [10], supplementation of organic acids was reported to decrease pH particularly in the crop, with some studies describing decreased pH up to the ileum. The organic acid formic acid, was reported to affect the microbial community and lead to increased performance and nutrient digestibility in broiler chickens [10]. Likely, the addition of formic acid to CaCO_3_-containing diets and the use of Ca-formate influences the microbial community in the digestive tract, possibly through changes in digesta pH [11, 12]. Therefore, it is possible that different microbiota composition is involved in causing the observed effects of Ca sources, organic acid supplementation, and Ca levels on P utilization.

The present study was conducted to investigate effects of diet acidification, dietary Ca, and phytase supplementation on the microbiota of the crop and the terminal small intestine of broiler chickens based on target amplicon sequencing of DNA, and to study consequences on pc InsP_6_ disappearance, and pc P and Ca digestibility. We used a 3 × 2 × 2 factorial design for the experiment (Table 1) with two Ca levels (5.6 and 8.2 g/kg dry matter) and two phytase supplementation levels (0 and 1500 FTU/kg). The third factor was the addition of 6 g formic acid/kg to CaCO_3_-containing diets or replacing CaCO_3_ by Ca-formate, which is termed “acidification” herein. By now, most studies only investigated one or two of these factors, making potential interactions between the three factors impossible to detect. This makes the present study the first to examine the influence of phytase and acidifying ingredients with different Ca levels on P utilization following InsP_6_ degradation, the microbial community, and potential linkages between all traits. To elucidate the underlying mechanisms, pH and inositol phosphate (**InsP**) isomers in the digesta of the crop, gizzard, and terminal small intestine were analyzed. It was hypothesized that replacing CaCO_3_ by Ca-formate or adding formic acid to CaCO_3_-containing diets decreases the pH, influences the microbial community, and increases InsP_6_ degradation and P digestibility.

**Table 1:** Description of 12 dietary treatments in a 3 × 2 × 2 experimental design

Acidification	Ca level (g/kg dry matter)	Phytase supplementation (FTU/kg)
CaCO_3_	5.6	0
		1500
	8.2	0
		1500
CaCO_3_ + formic acid	5.6	0
		1500
	8.2	0
		1500
Ca-formate	5.6	0
		1500
	8.2	0
		1500

## Results

The initial group weight at the beginning of feeding the experimental diets, on day 16 of life, was similar for all treatments, at 9604 g/pen (standard deviation = 15 g/pen, *P* = 0.183). The experimental period ended on day 21 and day 22 post-hatch for half of the replicate pens of each treatment. Mortality was low (0.6% of all animals) and not related to any treatment (six cases in six different treatments).

### Growth performance

**Fig. 1:**
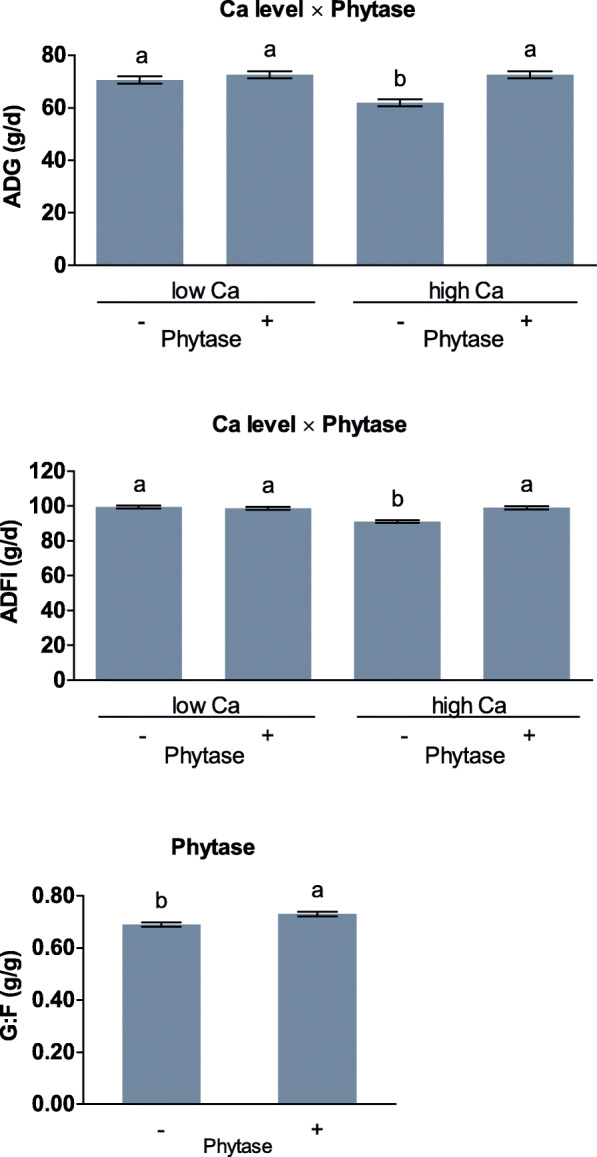
Average daily gain (ADG), average daily feed intake (ADFI), and the gain to feed ratio (G:F) of broiler chickens fed with differently acidified diets with low and high Ca levels and without (−) or with (+) supplementation of 1500 FTU phytase/kg fed from day 16 of life to the end of the experiment on day 21 or 22 for half of the replicate pens of each treatment. Only significant (*P* < 0.050) effects are shown. Columns within a statistical comparison not sharing the same letter are significantly different (*P* ≤ 0.050). Details of the statistical evaluations are shown in Table S1

The average daily gain (**ADG**) and average daily feed intake (**ADFI**) during the experimental period were lower compared to other treatments when the Ca level was high and no phytase was supplemented (*P* < 0.001; Fig. 1, Table S1). The ADFI was significantly lower for Ca-formate compared to CaCO_3_ and CaCO_3_ + formic acid (*P* = 0.002). Phytase supplementation increased the gain to feed ratio (**G:F**) by 0.04 g/g (*P* < 0.001). Further effects on growth performance were not significant.

### pH in the digestive tract

**Fig. 2:**
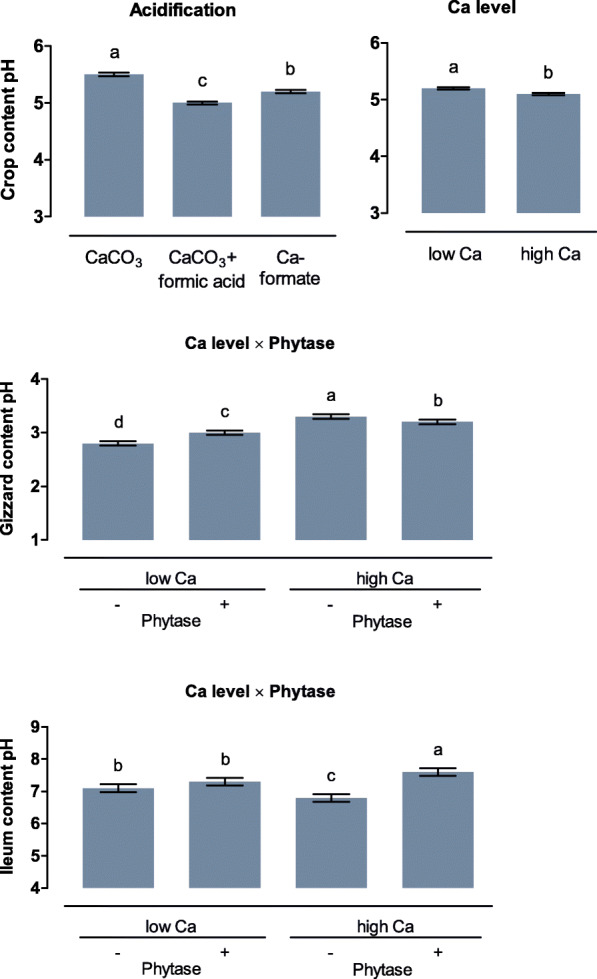
pH in the crop, gizzard, and ileum of broiler chickens fed with differently acidified diets with low and high Ca levels and without (−) or with (+) supplementation of 1500 FTU phytase/kg. Only significant (*P* < 0.050) effects are shown. Columns within a statistical comparison not sharing the same letter are significantly different (*P* ≤ 0.050). Details of the statistical evaluations are shown in Table S2

The contents of crop, gizzard, and posterior small intestine were obtained immediately after slaughter of the birds. In the crop content, the highest pH of 5.5 was observed for CaCO_3_. Crop pH was decreased by 0.6 and 0.3 units for CaCO_3_ + formic acid and Ca-formate, respectively, compared to CaCO_3_ (*P* < 0.001; Fig. 2, Table S2). With a 0.1-unit increase, crop pH was marginally but significantly higher for the high compared to the low Ca level. In the gizzard, phytase supplementation increased pH by 0.2 units (*P* < 0.001) and decreased pH by 0.1 units (*P* = 0.005) at the low and high Ca level, respectively. In the ileum, phytase supplementation increased pH by 0.8 units at the high Ca level (*P* < 0.001) but had no significant effect at the low Ca level.

### Microbial community

**Fig. 3:**
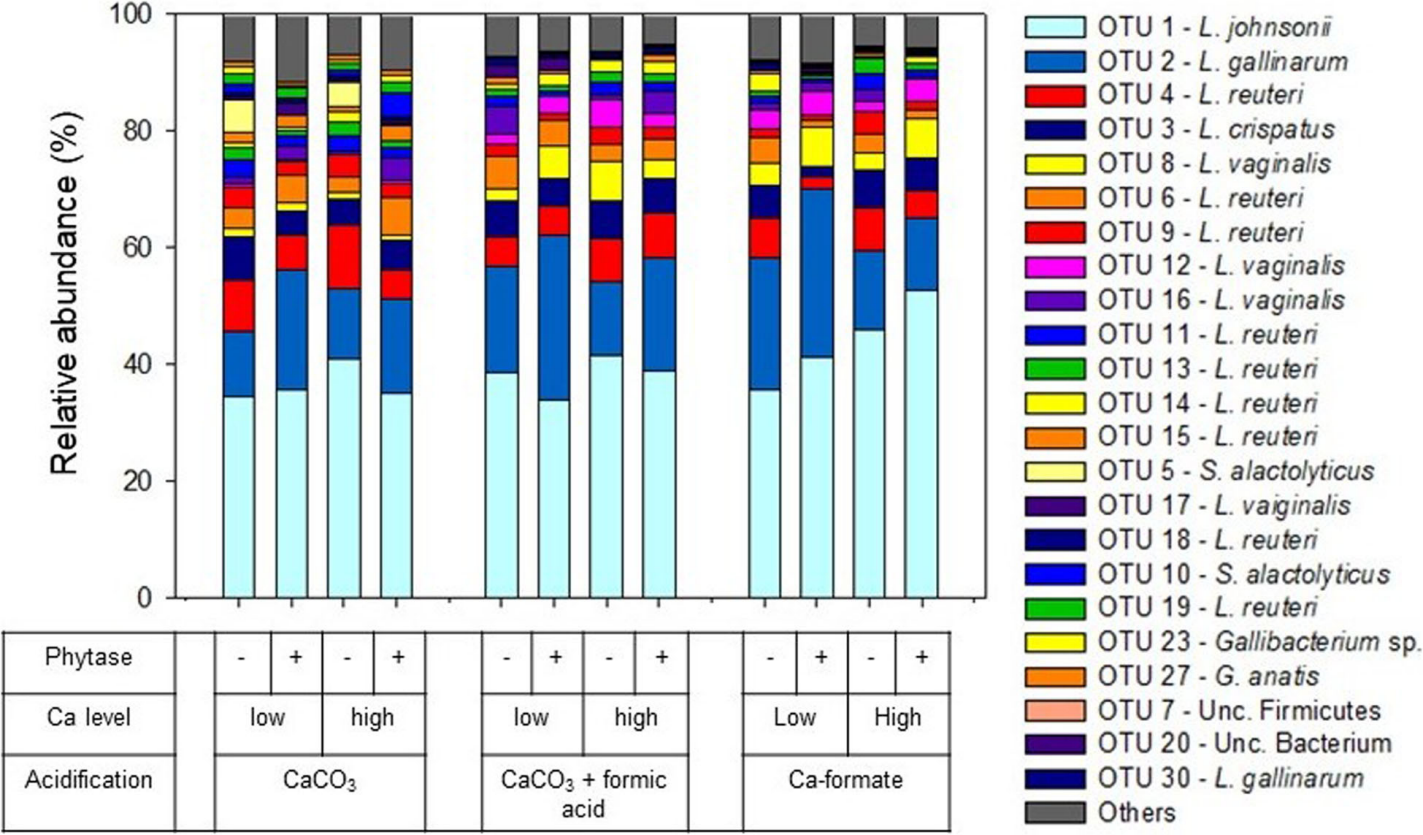
Relative abundance of OTUs in the crop content of broiler chickens in the respective dietary treatments (only OTUs with a relative abundance > 1% are presented)

**Fig. 4:**
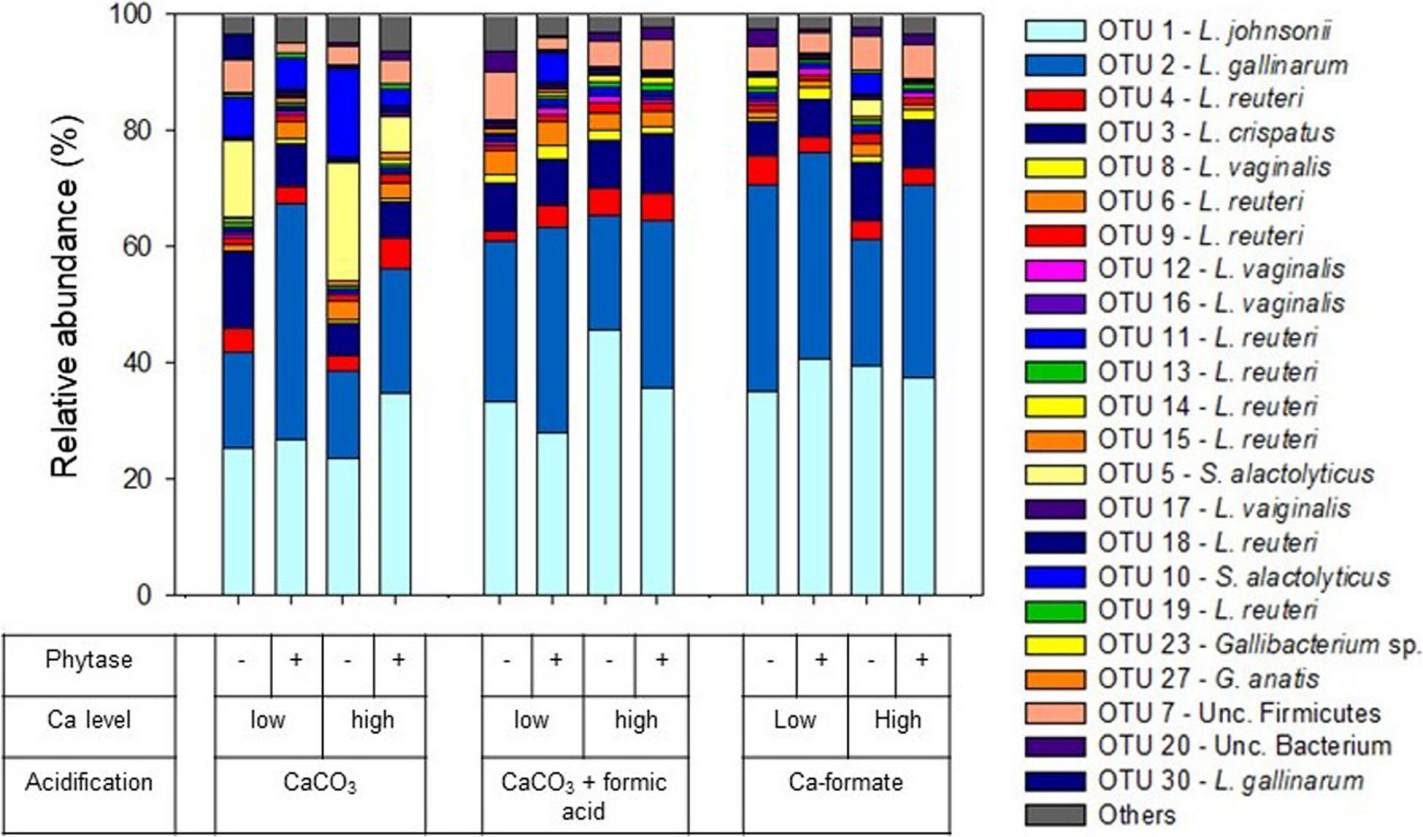
Relative abundance of OTUs in the ileum digesta of broiler chickens in the respective dietary treatments (only OTUs with a relative abundance > 1% are presented)

In content of both the crop and the ileum, mainly *Lactobacillus* species, including *L. johnsonii*, *L. crispatus, L. reuteri, L. gallinarum,* and *L. vaginalis*, were identified (Fig. 3 and Fig. 4). *Streptococcus alactolyticus* was highly abundant in the crop and the ileum for diets with CaCO_3_ at the low Ca level, irrespective of phytase supplementation. This was reflected by a high similarity between CaCO_3_ treatments at the low Ca level and a separation of these treatments from the other diets in a cluster analysis (Fig. S1). Permutational multivariate analysis of variance (**PERMANOVA**) analyses (Table S3) revealed that the microbial communities in the crop and the ileum were significantly affected by all the main effects (*P* ≤ 0.034), with no interaction being significant. The microbial community in birds receiving CaCO_3_ differed significantly in both sections of the digestive tract compared to CaCO_3_ + formic acid or Ca-formate (*P* ≤ 0.005).

In the crop content, between 34 and 53% of the detected relative abundance of microorganisms corresponded to operative taxonomic unit (**OTU**) 1 (*L. johnsonii*) and between 11 and 29% to OTU2 (*L. gallinarum*). We conducted analyses of variance (**ANOVA**) to get information on treatment effects on single OTUs being aware that explanatory power is partly impinged by physiological interrelationships between microorganisms. Abundance of OTU1 (*L. johnsonii*) was higher for Ca-formate when phytase was supplemented (*P* ≤ 0.050) (Table S4). The abundance of OTU1 increased for Ca-formate at the high Ca level. CaCO_3_ increased the abundance of the OTUs assigned to *L. reuteri* (OTU9, OTU11, OTU15, and OTU19, *P* ≤ 0.001). The high Ca level led to an increased abundance of OTU4 (*P* = 0.005), OTU9 (*P* = 0.036), and OTU11 (*P* < 0.001), which were assigned to *L. reuteri*. Phytase supplementation increased the abundance of OTU9, OTU11, and OTU13 (*L. reuteri*). Further significant influences on OTUs assigned to *L. vaginalis*, *L. gallinarum*, *S. alactolyticus*, *Gallibacterium sp.*, Unc. Bacterium, and Unc. Firmicutes are shown in Table S4 and Table S5. Crop pH was positively correlated with seven out of nine OTUs assigned to *L. reuteri* (*P* ≤ 0.027, Table S6, Fig. S2). Similar to the crop, the most abundant OTUs in the ileum were OTU1 (*L. johnsonii*, 24–46%) and OTU2 (*L. gallinarum*, 15–41%). Phytase supplementation increased the abundance of OTU1 (*P* = 0.040) and OTU2 (*P* = 0.015). The abundance of OTU2 was increased in the ileum of chicken fed with the high Ca level diets (*P* = 0.009). The abundance of OTUs assigned to *L. reuteri* (OTU6, OTU15, and OTU18, *P* ≤ 0.049) were decreased for Ca-formate and the abundance of OTU19 (*L. reuteri*, *P* = 0.049) was increased for CaCO_3_.

Six significant correlations (*P* ≤ 0.038) with OTUs assigned to *L. johnsonii*, *L. gallinarum*, and *L. reuteri* were determined for InsP_6_ concentration in the ileum, pc P digestibility, and pc InsP_6_ disappearance (OTU1, OTU2, OTU4, OTU9, OTU11, and OTU13) (Table S7, Fig. S3). Correlations with OTUs assigned to Unc. *Firmicutes*, *L. reuteri*, *S. alactolyticus*, and *Gallibacterium* sp. were significant in five cases for pH in the ileum (OTU7, OTU9, OTU10, OTU11, and OTU23). Correlations with concentrations of *myo*-inositol in the ileum were significant for OTUs assigned to *L. johnsonii*, *L. gallinarum*, and *L. reuteri* (OTU1, OTU2, OTU9, OTU11, and OTU13).

### Functional prediction

**Fig. 5:**
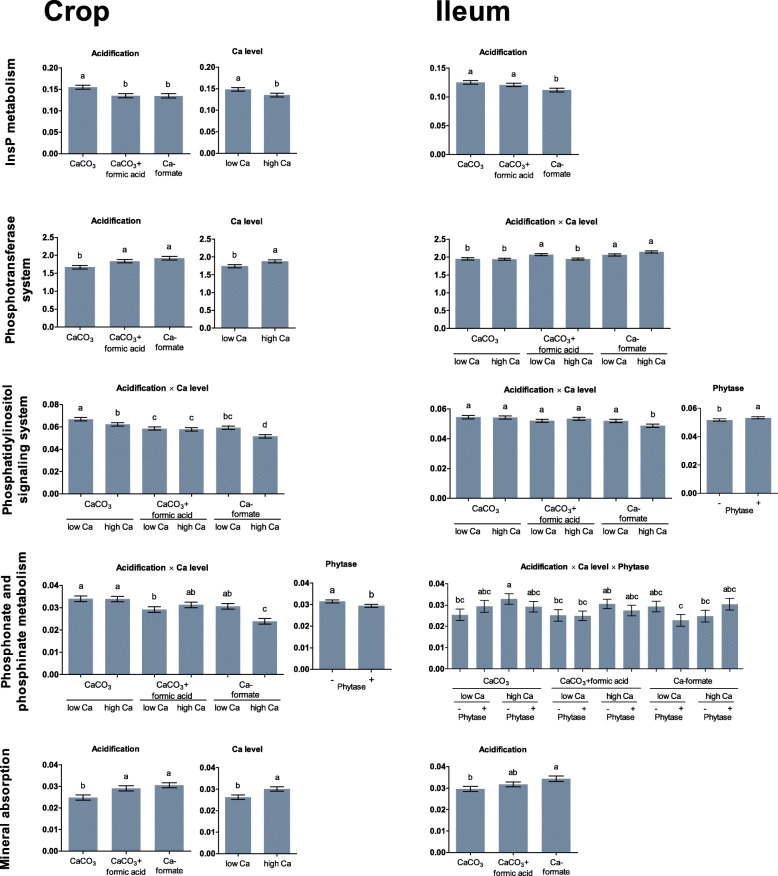
Relative proportion of genes assigned to P-related KEGG pathways in the crop content and the ileum digesta of broiler chickens fed with differently acidified diets with low and high Ca levels without (−) and with (+) supplementation of 1500 FTU phytase/kg. Only significantly influenced pathways are presented (*P* < 0.050). Columns within a statistical comparison not sharing the same letter are significantly different (*P* ≤ 0.050). Details of the statistical evaluations are shown in Table S8

The broad classification hierarchy of KEGG pathways of functions showed that same P-related pathways were significantly influenced in the crop and the ileum (Fig. 5, Table S8). No interaction related to genes connected to InsP metabolism was significant in the crop and the ileum. In the crop, genes encoding for InsP metabolism were higher in the CaCO_3_ than in the CaCO_3_ + formic acid and Ca-formate treatments (*P* ≤ 0.001) and more abundant at the low than at the high Ca level (*P* = 0.007). In the ileum, the abundance of genes related to InsP metabolism was lower in the Ca-formate treatment than in the CaCO_3_ and CaCO_3_ + formic acid treatments (*P* ≤ 0.011) and not influenced by Ca level. Phytase supplementation had no effect on InsP metabolism pathways in the crop and the ileum. Other significantly influenced pathways were mineral absorption, the phosphotransferase system, the phosphatidylinositol signaling system, and phosphonate and phosphinate metabolism in the crop and the ileum. Phytase supplementation decreased the abundances of genes related to the phosphonate and phosphinate metabolism (*P* = 0.048) in the crop and increased the abundances of genes related to the phosphatidylinositol signaling system in the ileum (*P* = 0.031). The other pathways were influenced by acidification, Ca level, or the two-way interaction between acidification and Ca level with no apparent pattern in changes.

**Fig. 6:**
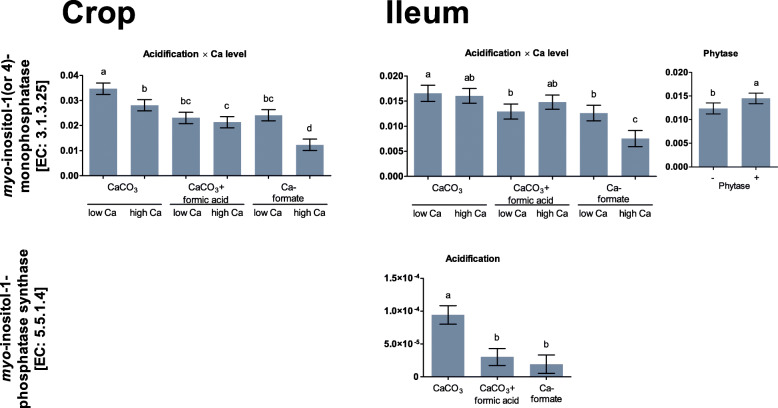
Relative proportion of genes assigned to enzymes related to inositol phosphate and myo-inositol degradation listed in the KEGG database in the crop content and the ileum digesta of broiler chickens fed with differently acidified diets with low and high Ca levels without (−) and with (+) supplementation of 1500 FTU phytase/kg. Only significantly influenced enzymes are presented (*P* < 0.050). Columns within a statistical comparison not sharing the same letter are significantly different (*P* ≤ 0.050). Details of the statistical evaluations are shown in Table S9

Within the category of InsP metabolism, increasing the Ca level reduced abundance of genes coding for *myo*-inositol-1(or 4)-monophosphatase in the CaCO_3_ and Ca-formate treatments (*P* ≤ 0.018), but not in the CaCO_3_ + formic acid treatment (*P* = 0.520) in the crop (Fig. 6, Table S9). In the ileum, *myo*-inositol-1(or 4)-monophosphatase was increased by phytase supplementation (*P* = 0.040). Increasing dietary Ca had no effect on *myo*-inositol-1(or 4)-monophosphatase in the CaCO_3_ and CaCO_3_ + formic acid treatments (*P* ≥ 0.269) and decreased *myo*-inositol-1(or 4)-monophosphatase in the Ca-formate treatment (*P* = 0.008). *Myo*-inositol-1-phosphate synthase coding genes were lower in the CaCO_3_ + formic acid and Ca-formate treatments than in the CaCO_3_ treatment in the ileum (*P* ≤ 0.001). Other genes annotated to InsP and *myo*-inositol degradation in the KEGG database were not influenced by the treatments used in this study.

### InsP_6_ disappearance and prececal digestibility of P and Ca

**Fig. 7:**
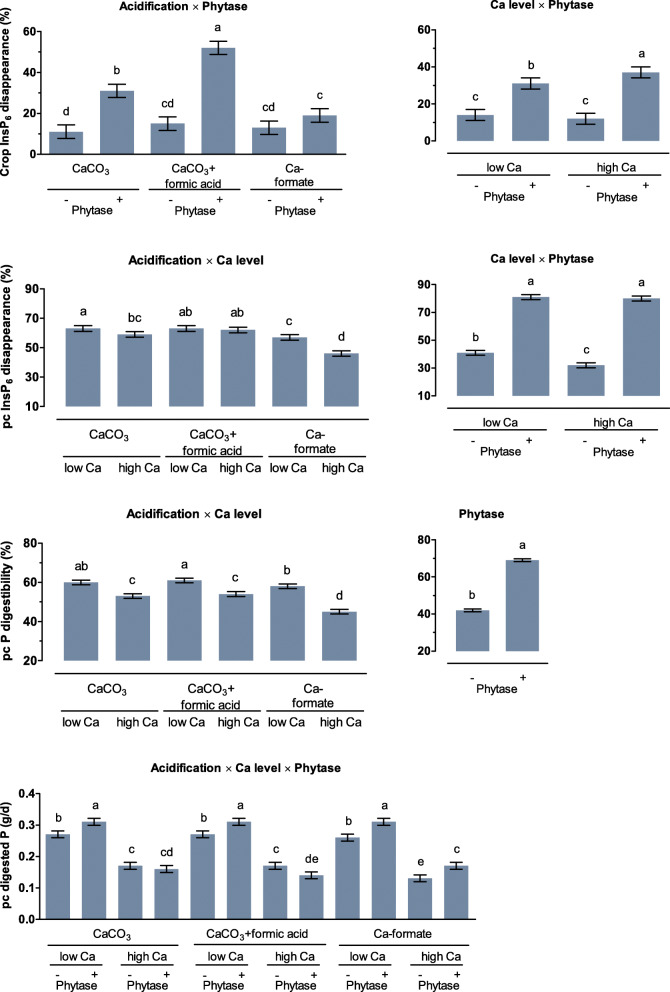
InsP_6_ disappearance in the crop, prececal (pc) InsP_6_ disappearance, pc P digestibility, and amount of pc digested P of broiler chickens fed with differently acidified diets with low and high Ca levels without (−) and with (+) supplementation of 1500 FTU phytase/kg. Only significant (*P* < 0.050) effects are shown. Columns within a statistical comparison not sharing the same letter are significantly different (*P* ≤ 0.050). Details of the statistical evaluations are shown in Table S10

Contents of crop, gizzard, and ileum were analyzed for InsP_6_ and degradation products, P, Ca, and titanium dioxide. InsP_6_ disappearance and mineral digestibility were calculated using titanium dioxide as the undigestible reference. In the crop, Ca level did not affect InsP_6_ disappearance (*P* = 0.536) when no phytase was supplemented (Fig. 7; Table S10). At the high Ca level, the effects of phytase supplementation on InsP_6_ disappearance in the crop increased by 6 percentage points (**pp**) to 37% (*P* = 0.025). Phytase supplementation increased InsP_6_ disappearance in the CaCO_3_ treatments with and without formic acid by 20 and 37 pp., respectively (*P* < 0.001), but not in the Ca-formate treatment (*P* = 0.090).

**Fig. 8:**
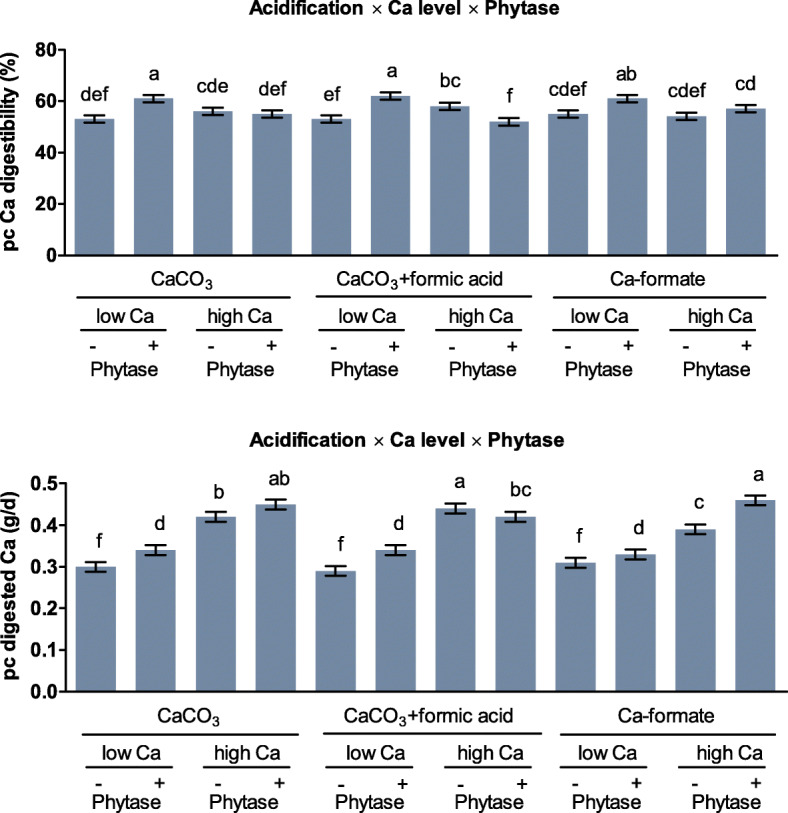
Prececal (pc) Ca digestibility and amount of pc digested Ca of broiler chickens fed with differently acidified diets with low and high Ca levels without (−) and with (+) supplementation of 1500 FTU phytase/kg. Only significant (*P* < 0.050) effects are shown. Columns within a statistical comparison not sharing the same letter are significantly different (*P* ≤ 0.050). Details of the statistical evaluations are shown in Table S10

Phytase supplementation increased pc InsP_6_ disappearance (*P* < 0.001) to approximately 80%, irrespective of the dietary Ca level. Without phytase supplementation, pc InsP_6_ disappearance was 9 pp. higher for the low compared to the high Ca level (*P* < 0.001). Ca level had no effect on pc InsP_6_ disappearance for CaCO_3_ + formic acid, but the high Ca level decreased pc InsP_6_ disappearance by 4 pp. for CaCO_3_ (*P* = 0.047) and by 10 pp. for Ca-formate (*P* = 0.016). Increasing dietary Ca decreased pc P digestibility. This effect was more pronounced for Ca-formate, with 12 pp. (*P* < 0.001), than for CaCO_3_ and CaCO_3_ + formic acid, with 7 pp. each (*P* < 0.001). The three-way interaction was significant for pc Ca digestibility (*P* = 0.012, Fig. 8). The pc Ca digestibility in CaCO_3_ and CaCO_3_-formate was increased by phytase supplementation at the low Ca level (*P* ≤ 0.012) but was not affected at the high Ca level (*P* ≥ 0.160). Phytase supplementation increased and decreased pc Ca digestibility for CaCO_3_ + formic acid at the low and high Ca level, respectively (*P* ≤ 0.002).

### *Inositol phosphate isomer and* myo*-inositol concentrations*

Significant interactions between acidification and phytase supplementation were detected for InsP_6_ and two InsP_5_ isomers in the crop (*P* < 0.001; Table S11). Decrease in concentrations of InsP_6_ and InsP_5_ isomers upon phytase supplementation was most pronounced in the CaCO_3_ + formic acid treatment and less in the CaCO_3_ and the Ca-formate treatments. *Myo*-inositol concentrations in the crop were not affected.

In the gizzard, InsP_6_ concentrations were lower in diets with supplemented phytase compared to those without (*P* < 0.001, Table S12). Highest InsP_6_ concentrations were found in the Ca-formate treatment without phytase supplementation. Phytase supplementation decreased concentrations of InsP_5_ isomers below level of detection, while making some InsP_4_ and InsP_3_ isomers detectable. Phytase supplementation increased *myo*-inositol concentrations to a greater extent at the low Ca level compared to the high Ca level (*P* = 0.022). Acidification had no effect on *myo*-inositol concentrations.

In the ileum, treatment effects on InsP_6_ concentration were inverse to those on pc InsP_6_ disappearance (Table S13). Phytase supplementation had no effect on Ins(1,2,4,5,6)P_5_ concentration, but increased Ins(1,2,3,4,5)P_5_ concentrations and decreased Ins(1,2,3,4,6)P_5_ concentrations in most cases. Concentrations of InsP_4_ and InsP_3x_ were increased upon phytase supplementation (*P* < 0.001). Ins(1,2,3,4,5)P_5_ concentrations were highest at the high Ca level for CaCO_3_ + formic acid and both Ca levels for Ca-formate when phytase was supplemented. Concentrations of Ins(1,2,3,4)P_4_ and InsP_3x_ were high when phytase was supplemented to CaCO_3_ + formic acid and Ca-formate at the high Ca level. An increase in *myo*-inositol concentrations upon phytase supplementation was more pronounced in the ileum at the low compared to the high Ca level. The highest *myo*-inositol concentration was determined for CaCO_3_ + formic acid (*P* ≤ 0.003) with no difference between CaCO_3_ and Ca-formate (*P* = 0.150).

## Discussion

Effects of phytase on InsP_6_ degradation and subsequent P utilization differ considerably between studies. Possible explanations include the use of acidifying ingredients in the diet and different Ca levels affecting the pH, phosphatase-producing bacteria, the probability that InsP_6_ complexes are formed, and the efficacy of phytases in the digestive tract. Most studies only investigated one factor, making potential interactions impossible to detect. This study first-time investigated influences of phytase and acidifying ingredients with different Ca levels and connects effects on the microbial community and P utilization following InsP_6_ degradation to find linkages between responses. We hypothesized that replacing CaCO_3_ by Ca-formate or adding formic acid to CaCO_3_-containing diets decreases the pH, influences the microbial community and its functionality, and increases InsP_6_ degradation and P digestibility.

### Crop

#### Crop microbiota and inositol phosphate degradation

In the crop, dietary treatments influenced both the microbial community and InsP_6_ degradation. This could have been independent effects or causal linkages. Adding formic acid to CaCO_3_ or replacing CaCO_3_ with Ca-formate decreased pH in the crop content and concurrently shifted microbiota composition. A connection between pH and abundance of OTUs is indicated by correlations (Table S4) but it is not clear whether pH or other consequences of Ca level and acidification were causative. The microbial communities of the CaCO_3_ + formic acid and Ca-formate treatments differed from that of the CaCO_3_ treatment, while no difference of the microbial community was determined between the CaCO_3_ + formic acid and Ca-formate treatments. Changes in pH were not completely concomitant with changes in the microbial community, suggesting that changes in the microbial community were caused by at least one other mechanism in addition to pH reduction. Likely, different mechanisms had an impact on the microbial community when the pH was reduced from 5.5 to 5.2 (CaCO_3_ to Ca-formate) and from pH 5.2 to 4.9 (Ca-formate to CaCO_3_ + formic acid). The microbial community may also have been affected by formic acid because the applied inclusion of formic acid is known to inhibit certain bacterial species including salmonella [13–15] and can be used as a carbon source by other bacteria [16].

Functional prediction and changes in the relative abundance of some bacteria may suggest an influence of the microbial community on P utilization. Phosphatase activity has been described for microorganisms, including *L. johnsonii* [17] and strains of *L. reuteri* [18]. Relative abundance of *L. johnsonii* ranged from 34 to 53% and depended on acidification and Ca level (Table S12). Hence, treatments probably influenced the contribution of *L. johnsonii* to P utilization and affected other traits of *L. johnsonii*; probiotic characteristics are assigned to *L. johnsonii*, such as reduced adhesion to the epithelial cells and inhibited growth of pathogenic microorganisms in humans [19], and they are further known to positively influence measures related to the immune system of broilers [20]. Relative abundance of OTUs assigned to strains of *L. reuteri* summed up to a range of 6–26%. At the low Ca level, the relative abundance of some OTUs assigned to *L. reuteri* (OTU4, OTU9, OTU11, and OTU13) were less abundant in the crop, which corroborates the finding that Ca can inhibit the growth of *L. reuteri* [18]. Hayek et al. [18] found that phosphatase activity (probably including phytase) produced by *L. reuteri* depended on strain and presence of chemical elements in a nutrient solution. Ca increased phosphatase activity of all *L. reuteri* strains under investigation. This effect was described to be caused by Ca-dependent activation of active sites of phosphatases, including phytase [21, 22]. In the present study, it appears possible that lower abundance of most *L. reuteri* strains were compensated by a higher potential to produce phosphatases by *L. reuteri* and other microorganisms at the higher Ca level when phytase was supplemented. Irrespective of individual bacteria strains, the InsP metabolism and other P-related metabolic pathways of the microbial community in the crop were influenced by acidification and Ca level. Within InsP metabolism, acidification and Ca level influenced the abundance of genes connected to enzymes involved in degradation of lower InsP isomers and *myo*-inositol. This strongly indicates that the microbial community contributes to InsP degradation in the crop; however, the extent of this contribution cannot be sufficiently quantified with the available data.

#### Effect of pH on InsP_6_ disappearance in the crop

**Fig. 9:**
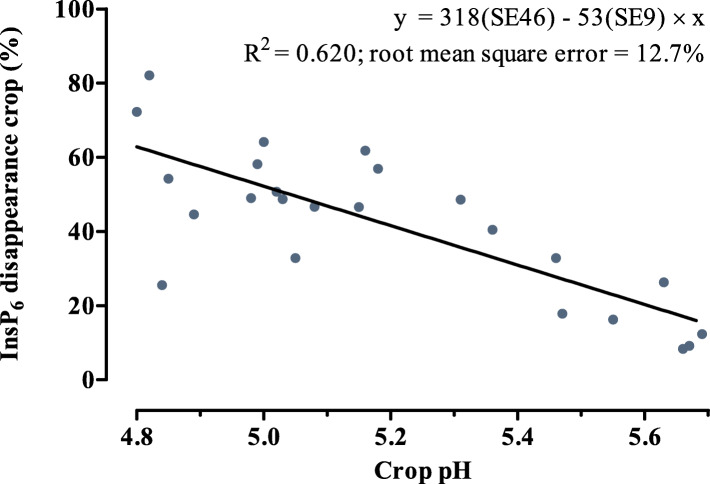
Relationship between pH and InsP_6_ disappearance in the crop of broiler chickens fed diets with CaCO_3_ (without and with formic acid) at two dietary Ca levels with supplementation of 1500 FTU phytase/kg. Dots represent observations corrected for block effects

Acidification and Ca level effects were observed in diets with phytase supplementation only. With a value of 55%, the highest InsP_6_ disappearance in the crop was observed when phytase was supplemented to CaCO_3_ + formic acid. This effect seems to have been caused by acidification of the crop content by formic acid, which then shifted the pH closer to the optimum of the phytase used (pH optimum of 4.5 according to the product datasheet of the producer). This explanation is supported by a linearly negative relation between crop pH and InsP_6_ disappearance in the phytase-supplemented diets (Fig. 9). The regression indicates that InsP_6_ disappearance in the crop was increased by 5.3 pp. per each 0.1-unit reduction of pH in the crop.

#### Ca solubility effects

Different Ca solubility seems to have an influence on InsP_6_ disappearance in the crop, but clear inferences are difficult to make. Higher solubility can make free Ca cations more available for Ca-InsP_6_ complex formation and, hence, reduce InsP_6_ disappearance. Ca solubility of Ca-formate was reported to be substantially higher than that of CaCO_3_ [23]. InsP_6_ disappearance in the crop was higher for CaCO_3_ compared to Ca-formate, consistent with the theory of higher Ca-InsP_6_ complex formation as caused by higher solubility of Ca-formate. This is supported by the crop pH of 5.2 for Ca-formate and 5.5 for CaCO_3_, both being above the pH critical for complex formation [9]. As acidification can increase Ca solubility, CaCO_3_ + formic acid probably had higher Ca solubility than CaCO_3_ alone as a consequence of formic acid lowering the pH [24]. Formic acid supplementation would then make more Ca available for Ca-InsP_6_ complex formation and thus reduce InsP_6_ disappearance. However, higher InsP_6_ disappearance in the crop was observed for CaCO_3_ + formic acid than for CaCO_3_. When Ca-formate was used instead of CaCO_3_, InsP_6_ disappearance in the crop was possibly determined more by the higher Ca-InsP_6_ complex formation caused by higher Ca solubility than by the lower pH. With formic acid supplementation, acidification may have had a higher impact on InsP_6_ degradation than the higher Ca solubility.

### Ileum and gizzard

#### Ileum microbiota and inositol phosphate degradation

The reducing effect of the high Ca level on pc InsP_6_ disappearance for CaCO_3_ but not for CaCO_3_ + formic acid may be caused by changes in endogenous phytase and other phosphatases produced by epithelial cells or by microbiota. The latter is supported by the effects of the dietary treatments on metabolic pathways of the microbial community connected to InsP_6_ degradation. Changes in P-related metabolic pathways in the ileum were similar to those in the crop. The composition of the microbial community varied among treatments. ANOVA analyses of relative abundances of single OTUs revealed significant interactions for five OTUs (1, 5, 7, 8, and 20; Table S5). However, an assessment of the contribution of these microorganisms to InsP degradation is difficult to derive because the phosphatase activity of the associated microorganisms is either not yet described in the literature or the OTUs define only classes of strains. Therefore, it cannot be estimated whether these microorganisms contributed phosphatase to a relevant extent. Nonetheless, results indicated that microorganisms in the digestive tract have contributed to P utilization of the birds. There were five positive correlations between pc P digestibility and OTUs assigned to *L. reuteri* and *L. johnsonii*; these microorganisms are known to produce phosphatase [17, 18]. Another possible explanation for differences in the microbial community could be an alteration of availability of nutrients for the microbiota in the digestive tract as a result of the varied supplements. To our knowledge, there is no literature available that supports or excludes one of these explanatory approaches.

#### Relation between calcium solubility, pH, and inositol phosphate degradation

High dietary Ca levels reduced pc InsP_6_ degradation, but this effect was compensated by phytase supplementation. A decline in pc InsP_6_ disappearance with graded levels of CaCO_3_ in diets for broiler chickens was previously described [3]. In the present study, effects of acidification, Ca level, and phytase supplementation on InsP_6_ degradation were interrelated. In contrast, Li et al. [7] and Dersjant-Li et al. [25] did not find an interaction between CaCO_3_ level and phytase supplementation on pc InsP_6_ disappearance and pc P digestibility when up to 1000 and 500 phytase units (**FTU**) phytase/kg were supplemented, respectively. However, dietary Ca levels in these studies were higher than in the present study and a different phytase product with different features such as pH optimum was used. We are not aware of other studies on InsP_6_ degradation that compared CaCO_3_ with the more soluble Ca-formate. Hamdi et al. [4] determined no significant difference in pc P digestibility when 5.5 g Ca/kg was supplied by CaCO_3_ or the more soluble Ca chloride. In another series of experiments, CaCO_3_ was compared with a calcified marine seaweed considered a highly soluble Ca source [26–28]. Results of this series were not consistent, likely owing to different Ca and phytase supplementation levels. Further, comparisons between calcified marine seaweed and Ca sources investigated in these studies may be impeded by unknown substances contained in the seaweed products. Overall, it seems that one major factor of P utilization affected by Ca sources and levels is the solubility of Ca from differing sources that depends, inter alia, on pH and microbiota. This supports conclusions drawn by Kim et al. [24] based on a comparison of CaCO_3_ sources.

**Fig. 10:**
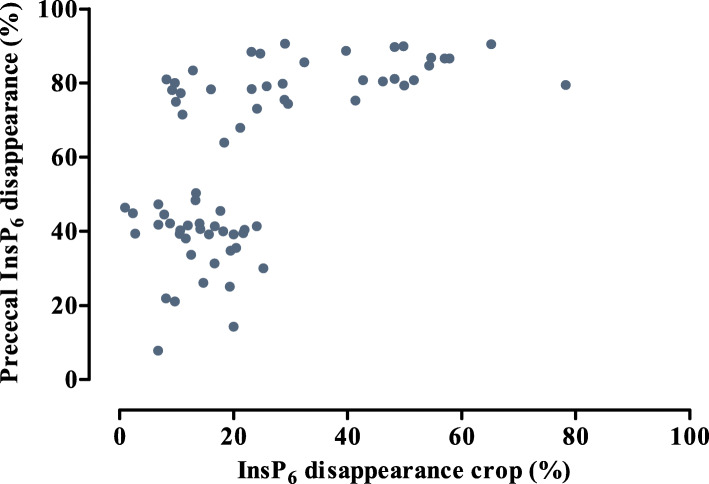
Relationship between InsP_6_ disappearance in the crop and prececal InsP_6_ disappearance of broiler chickens. Dots represent observations corrected for block effects without (below 60% prececal InsP6 disappearance) or with (above 60% prececal InsP_6_ disappearance) supplementation of 1500 FTU phytase/kg

Differences in pc InsP_6_ disappearance between CaCO_3_, CaCO_3_ + formic acid, and Ca-formate were smaller compared to InsP_6_ disappearance in the crop. A clear determination of pc InsP_6_ disappearance as compared to InsP_6_ disappearance in the crop has been found in the present study, irrespective of whether phytase was supplemented or not (Fig. 10). This indicates the cleavage of Ca-InsP_6_ complexes due to decreasing pH in the proventriculus and the gizzard. Gizzard pH ranged from 2.8–3.3, which was probably sufficient for cleavage of Ca-InsP_6_ complexes because Ca-InsP_6_ complexes occur at pH 4 and higher [29].

No difference in pc InsP_6_ disappearance was found between CaCO_3_ + formic acid at both Ca levels and the lower Ca level of CaCO_3_. A reason for this could be the higher efficacy of phytase caused by a decreased crop pH and less complete InsP_6_ degradation for Ca-formate due to higher Ca solubility. We are not aware of other studies investigating a simultaneous supplementation of formic acid and phytase in poultry. However, in pigs, total tract P digestibility was increased to a higher extent when phytase and formic acid were supplemented together than when their separate effects were summed [30].

#### Effects on inositol phosphate degradation pathways

Concentrations of lower InsP isomers in gastrointestinal content give insights into effects on InsP degradation pathways, including limiting steps in the process of InsP degradation. InsP isomers that limit InsP degradation apparently differed among treatments. When no phytase was supplemented, InsP degradation by endogenous enzymes seems to have been limited by the high Ca level. High Ca supposedly inhibited the activity of endogenous enzymes at different stages of the degradation pathway of InsP_6_ and lower InsP isomers depending on the Ca source. This interpretation is based on lower concentrations of *myo*-inositol and higher concentrations of InsP_6_ and InsP_5_ isomers in the ileum at the high compared to the low Ca level of the CaCO_3_ and Ca-formate treatments. In the CaCO_3_ + formic acid treatment, there was no difference in InsP_6_ concentrations in the ileum between the Ca levels, but there was slightly higher InsP_5_ isomer and lower *myo*-inositol concentrations at the high Ca level. Obviously, formic acid supplementation overcame a restricted release of the first phosphate group from InsP_6_ at the high Ca level that was observed in the CaCO_3_ and Ca-formate treatments.

Degradation of lower InsP isomers in the ileum seems to be impaired by the high Ca level when phytase was supplemented. In the CaCO_3_ treatment_,_ higher InsP_4_ and InsP_3_ isomer concentrations and lower *myo*-inositol concentrations were found for the high compared to the low Ca level. This suggests a diminishing effect of a higher Ca level on phosphatases degrading InsP_4_ and lower InsP isomers. Similar InsP_3_ to InsP_6_ concentrations were determined between the low Ca level of the CaCO_3_ and the CaCO_3_ + formic acid treatments, but higher *myo*-inositol concentrations were determined for the CaCO_3_ + formic acid treatment. This may indicate a higher or faster hydrolysis of InsP_2_ and InsP_1_ isomers for the CaCO_3_ + formic acid treatment at the low Ca level. A fast degradation of InsP_2_ and InsP_1_ isomers by the use of formic acid could also be observed in the higher Ca level. At high Ca levels, higher InsP_5_ to InsP_3_ concentrations were found for the CaCO_3_ + formic acid treatment compared to the CaCO_3_ treatment, but there was no difference in *myo*-inositol concentration. Despite a slower degradation of InsP_5_ to InsP_3_ isomers, this points towards a high hydrolysis of InsP_2_ and InsP_1_ isomers for the high Ca level for CaCO_3_ + formic acid. However, it should be of greater interest to degrade InsP with higher phosphorylation first so as to diminish potential chelating effects. Comparing the low Ca levels in the CaCO_3_ and Ca-formate treatments, higher concentrations of InsP_6_ and InsP_5_ isomers and similar *myo*-inositol concentrations were found, indicating that the initial steps of InsP_6_ degradation were impeded for Ca-formate, and degradation of InsP isomers lower than InsP_5_ was not the limiting factor. Degradation of InsP_2_ and InsP_1_ isomers seems to be particularly relevant for InsP degradation at both Ca levels for CaCO_3_ + formic acid when phytase was supplemented. This makes the knowledge of InsP_2_ and InsP_1_ isomer concentrations necessary so as to determine InsP isomers that limit InsP degradation.

#### Effects on calcium digestibility

Responses in pc Ca digestibility differed from other traits under investigation, probably as a result of highly regulated Ca homeostasis and regulation of Ca absorption [31]. Phytase supplementation increased the pc digested amount of Ca irrespective of acidification at the low Ca level, whereas such an effect was only observed for Ca-formate at the high Ca level. This may be explained by a maximum pc digested Ca amount of about 0.42 g/d. This amount was met at the high Ca level without phytase supplementation for CaCO_3_ and CaCO_3_ + formic acid, but for Ca-formate only when phytase was supplemented. Particle size [28, 32] and Ca solubility [24] of Ca sources were shown to be a determinant of Ca utilization. Possibly, the considerably higher mean particle size of Ca-formate compared to CaCO_3_ was more limiting for Ca utilization than the higher solubility of Ca-formate.

## Conclusions

Replacing CaCO_3_ by Ca-formate and adding formic acid to CaCO_3_-containing diets decreased digesta pH, influenced the microbial community, and had an effect on InsP_6_ degradation. Results imply that InsP degradation in the crop and until the terminal ileum may partly be explained by the microbial community because relationships between InsP degradation and relative abundance of phosphatase-producing strains *L. johnsonii* and *L. reuteri* were observed. Functional prediction also suggested influences of the microbiota on InsP degradation. In addition to microbiota, Ca effects on InsP degradation and pc P digestibility were shown to depend on dietary concentration, solubility of the Ca sources, as well as on the consequences of Ca supply on the pH of the digestive tract. The results of this study are relevant to the industry because choice of concentration and source of Ca in the diet is an important factor of feed formulation that affects P utilization by the animals.

## Methods

### Animals and management

The experiment was conducted at the Agriculture Experiment Station of the University of Hohenheim. Unsexed Ross 308 broilers were obtained from a commercial hatchery (Brüterei Süd ZN der BWE-Brüterei Weser-Ems GmbH & Co. KG, Regenstauf, Germany). The hatchlings were placed in 72 floor pens (115 × 230 cm ground area, 260 cm height) in groups of 15. The temperature was gradually reduced from 34 °C at placement to 26 °C at the end of the experiment on day 22. The light regimen was 24:0 h of light:dark in the first three days and 18:6 h of light:dark from day 4 until the end of the experiment. Birds were kept on wood shavings for the first 15 days. On day 16, the litter was removed from the floor and birds were kept on perforated floors thereafter. Birds were reallocated among pens on day 16 to achieve a similar group weight in all pens. The pens were randomly assigned to the treatments in a completely randomized block design. Feed and water were available for ad libitum consumption throughout the experiment.

### Diets

A commercial starter diet was provided for the first 15 days and contained, per kg, 215 g crude protein, 11 g Ca, 7 g P, 12.5 MJ metabolizable energy, 110 mg monensin sodium, 10 IU endo-1,4-β-xylanase, and 750 FTU of a 6-phytase (Deutsche Tiernahrung Cremer GmbH & Co. KG, Düsseldorf, Germany). From day 16, the experimental diets were provided.

Twelve experimental diets were mixed (Table S14). Except for Ca and P, the diets were calculated to meet or exceed the supply recommendations of the Gesellschaft für Ernährungsphysiologie [33]. The diets were based on corn, soybean meal, rapeseed meal, and sunflower meal and were formulated without mineral P. High levels of oilseed meals were included to achieve high InsP_6_ concentrations as a substrate for the added phytase. Titanium dioxide was included as an indigestible marker at a level of 5 g/kg. Diets contained CaCO_3_, CaCO_3_ + formic acid (6 g/kg; Amasil 85, BASF SE, Germany; > 85% wt/wt formic acid), or Ca-formate. CaCO_3_ and Ca-formate were added in two concentrations in order to achieve dietary Ca levels of 5.6 g Ca/kg dry matter (“low”) or 8.2 g Ca/kg dry matter (“high”). One half of each diet was supplemented with 1500 FTU phytase/kg (“+”; Natuphos E 5000 G, BASF SE, Germany). The other half remained without phytase supplement (“-”). Diamol (diatomaceous earth) was used to balance mass differences between diets. Overall, analyzed nutrient concentrations confirmed calculated values (Table S15). The diets were produced by Research Diet Services (Research Diet Services BV, Hoge Maat 10, 3961NC, Wijk bij Duurstede, Netherlands) and pelleted through a 3-mm die.

### Measurements and sampling procedures

Animals and feed were weighed on day 16 and at the end of the experimental period on a pen basis. At the end of the experiment, half of the pens of each treatment were processed on day 21 and day 22, respectively, for capacity reasons. The animals were deprived of feed 2 h before slaughter, and, feeders were moved back into the pens 1 h before slaughter in order to standardize gut fill. The animals were stunned using a gas mixture (35% CO_2_, 35% N_2_, and 30% O_2_) and euthanized by CO_2_ exposure. Crop and gizzard content was removed with a spatula without scraping the mucosa and pooled on a pen basis. A subsample of the pooled crop digesta was used for microbiota analysis and pH measurement. The section between Meckel’s diverticulum and 2 cm anterior to the ileo-ceco-colonic junction, herein defined as the ileum, was removed. Digesta from the posterior half of the ileum was sampled because P absorption in the anterior third of the ileum may not have been completed [34]. Approximately 2 cm of this ileum section was used for pH and microbiota analysis. For this purpose, the digesta was carefully stripped out. Samples for microbiota analyses were immediately stored at − 20 °C. The digesta of the remainder of this ileum section was flushed out using ice-cold deionized water and pooled on a pen basis. Digesta samples were immediately frozen at − 20 °C until they were freeze-dried.

### Microbial community analyses

DNA from crop and ileum digesta samples were extracted with the commercial DNA extraction kit FastDNA™ Spin Kit for soil (MP Biomedicals LLC, Solon, OH, USA). DNA was further quantified with a NanoDrop 2000 spectrophotometer (Thermo Fisher Scientific, MA, USA) and stored at − 20 °C. Illumina library was prepared according to Kaewtapee et al. [35]. The V1–2 region of the 16S rRNA gene was amplified and 1 μl of the first polymerase-chain reaction (**PCR**) product was used as a template in the second PCR with multiplexing and indexing primers as described previously [36]. Samples were sent for pair-end sequencing using the 250 bp paired-end sequencing chemistry on an Illumina MiSeq platform. Raw reads were checked for quality, assembled, and aligned using the mothur pipeline tool [37]. The data included 74,662 ± 3399 sequences per sample. The UCHIME program included in the mothur pipeline was used to identify possible chimeras [38]. Reads were clustered at 97% identity into 681 OTUs. Only OTUs with an average abundance higher than 0.0001% and a sequence longer than 250 bp were considered for further analysis. The closest representative was manually identified using seqmatch from the Ribosomal Database Project [39]. Sequences were submitted to European Nucleotide Archive under the accession number PRJEB38378.

Prediction of functionality was carried out with the R package Tax4Fun2 [40], which relied on the SILVA database [41] and used the KEGG hierarchy, comprising of gene catalogs from sequenced genomes [42], for the assignations. To assign functionality, the BIOM table was obtained from the mothur pipeline [43]. Genomes from 16S rRNA gene sequences identified in this study were downloaded from the NCBI database (https://www.ncbi.nlm.nih.gov/home/genomes/) in order to produce a database specially made for the crop and ileum of chickens.

### Chemical and physical analyses

Samples of all diets were pulverized using a vibrating cup mill (Pulverisette 9, Fritsch GmbH, Idar-Oberstein, Germany) for chemical analyses of gross energy, P, Ca, Ti, InsP isomers, and *myo*-inositol, or ground to pass through a 0.5 mm sieve (Ultra Centrifugal Mill ZM 200, Retsch GmbH, Haan, Germany) for all other analyses. Digesta samples were pulverized using the same vibrating cup mill. Ground samples were analyzed for proximate nutrients and fiber fractions according to the methods of Verband Deutscher Landwirtschaftlicher Untersuchungs- und Forschungsanstalten [44]. The concentrations of Ti, P, and Ca in pulverized feed and digesta samples were analyzed using inductively coupled plasma-optical emission spectrometry following wet digestion [45]. InsP_6_ and InsP_3–5_ isomers were analyzed in pulverized feed and digesta samples according to methods described by Zeller et al. [45] with modifications noted by Sommerfeld et al. [1]. Separation of enantiomers is not possible using this methodology; therefore, we were unable to distinguish between the d- and l-forms. Some InsP_3_ isomers could not be identified because standards were unavailable. Clear discrimination of the isomers Ins (1,2,6) P_3_, Ins (1,4,5) P_3_, and Ins (2,4,5) P_3_ was not possible because they co-eluted, and therefore the term InsP_3x_ was used for these InsP_3_ isomers of unknown proportions. *Myo*-inositol in feed and digesta samples was analyzed according to Sommerfeld et al. [46], using gas chromatography/mass spectrometry following derivatization. Phytase activity of the diets was analyzed according to an ISO standard method [47]. Measurements of pH in the undiluted content of the digestive tract were done using a CG 840 digital pH meter (Schott-Geräte GmbH, Mainz, Germany) equipped with a temperature probe and a puncture solid-state pH electrode suitable for semi-solid samples (InLab Solids Pro-ISM, Mettler Toledo Inc., Columbus, USA) as described previously [48].

Particle size distribution of CaCO_3_ and Ca-formate were analyzed at Forschungsinstitut Futtermitteltechnik e.V. Braunschweig, Germany by laser diffraction (Sensor: HELOS Hi202, measuring range: 0.5/0.9–175 μm, dispersing system: Rodos/L (402F); SYMPATEC, Clausthal-Zellerfeld). Particle sizes of the diets were measured as described by Grubješić et al. [49] by wet sieving analysis using a sieve shaker (AS200, Retsch GmbH, Germany) with sieve sizes of 2, 1.18, 1, 0.5, 0.25, 0.125, and 0.063 mm.

### Calculations and statistics

The ADG, ADFI, and G:F were calculated on a pen basis from day 16 to the end of the experiment and corrected for mortality. Prececal InsP_6_ disappearance and pc digestibility of P and Ca (y) were calculated on a pen basis using the following equation:

1 $$ \mathrm{y}\left(\%\right)=100-100\times \left(\frac{Ti{O}_2 infeed\left(g/ kgdrymatter\right)}{Ti{O}_2 indigesta\left(g/ kgdrymatter\right)}\times \frac{Analyteindigesta\left(g/ kgdrymatter\right)}{Analyteinfeed\left(g/ kgdrymatter\right)}\right) $$

Statistical evaluation of all traits was performed according to the following model:

2 $$ {\mathrm{y}}_{ijkl}=\mu +{\mathrm{Acidification}}_i+\mathrm{Ca}{\mathrm{level}}_j+{\mathrm{Phytase}}_k+\left({\mathrm{Acidification}}_i\times \mathrm{Ca}{\mathrm{level}}_j\right)+\left({\mathrm{Acidification}}_i\times {\mathrm{Phytase}}_k\right)+\left(\mathrm{Ca}{\mathrm{level}}_j\times {\mathrm{Phytase}}_k\right)+\left({\mathrm{Acidification}}_i\times \mathrm{Ca}{\mathrm{level}}_j\times {\mathrm{Phytase}}_k\right)+{\mathrm{block}}_l+{\mathrm{e}}_{ijkl} $$

where y_*ijkl*_ is the mean value of each treatment; μ is the mean of all treatments; Acidification_*i*_ is the fixed effect of diets containing CaCO_3_, CaCO_3_ + formic acid, or Ca-formate; Ca level_*j*_ is the fixed effect of the Ca level (5.6 or 8.2 g/kg dry matter); Phytase_*k*_ is the fixed effect of phytase supplementation (0 or 1500 FTU phytase/kg); block_*l*_ is the random block effect; and e_*ijkl*_ is the residual error. The block effect included possible effects of location in the building and sampling time on day 21 or day 22 because three blocks were sampled each day. ANOVA was calculated using the MIXED procedure of the SAS for Windows (version 9.4, SAS Institute, Cary, NC, USA). Normal distribution and homogeneity of variance were tested prior to statistical analysis. *P* values described herein result from ANOVA, or *t* tests when two groups were compared.

In order to describe the particle size distribution of the diets, the equation described by Siegert et al. [50] was fitted to the results of the sieve analysis using the NLMIXED procedure of SAS:

3 $$ y=\frac{100}{1+{e}^{\left(-a\times \left(x-b\right)\right)}} $$

where y is the cumulative percentage of particles smaller than the sieve size x (mm), a is the slope of the regression, and b is the inflection point, which can be interpreted as the mean particle size (mm).

The sequencing dataset was statistically analyzed as described by Borda-Molina et al. [51] using the PRIMER software (PRIMER-E, version 7.0.9, Plymouth Marine Laboratory, Plymouth, UK) [52]. The dataset was first standardized by the total, then comparisons between samples were made through a sample similarity matrix using the Bray-Curtis coefficient algorithm. A hierarchical cluster analysis was done to show the similarity between samples. PERMANOVA was used to compare the microbial community among the treatments and between the sections (PRIMER-E, version 7.0.9, Plymouth Marine Laboratory, Plymouth, UK). The similarity percentage analysis (**SIMPER**) identified the OTU contribution to the similarity among samples within each treatment. Differences in the relative abundance of single OTUs were also estimated based on eq. 2. Pearson correlations with other traits were calculated for OTUs with a relative abundance > 1% using GraphPad Prism 5 (GraphPad Software Inc., San Diego, CA, USA). Significance was declared at *P* ≤ 0.050 for all statistical analyses.

## Supplementary Information

**Additional file 1: Table S1.** Growth performance. **Table S2.** Crop content and ileum digesta pH. **Table S3.** PERMANOVA of the microbial community in crop content and ileum digesta. **Table S4.** Relative abundance of OTUs in the crop content. **Table S5.** Relative abundance of OTUs in the ileum digesta. **Table S6.** Correlation between OTUs in the crop content and other measured traits. **Table S7.** Correlation between OTUs in the ileum content and other measured traits. **Table S8.** Influences on genes assigned to P-related pathways in crop content and ileum digesta. **Table S9.** Influences on genes assigned to enzymes related to inositol phosphate and *myo*-inositol degradation in crop content and ileum digesta. **Table S10.** InsP_6_ disappearance and prececal P digestibility. **Table S11.** InsP_6_, lower inositol phosphate isomers, and *myo*-inositol in the crop content. **Table S12.** InsP_6_, lower inositol phosphate isomers, and *myo*-inositol in the gizzard digesta. **Table S13.** InsP_6_, lower inositol phosphate isomers, and *myo*-inositol in the ileum digesta. **Table S14.** Composition of the experimental diets. **Table S15.** Analyses of experimental diets. **Figure S1.** Cluster analysis similarity in crop content and ileum digesta. **Figure S2.** Relationship crop pH and relative abundance of OTU2 in the crop content. **Figure S3.** Relation between the relative abundances of OTU1 and OTU2 with other measured traits.

## Data Availability

All data generated or analyzed during this study are included in this published article and its supplementary information files.

## Abbreviations

ADFI: Average daily feed intake
ADG: Average daily gain
ANOVA: Analysis of variance
Ca: Calcium
CaCO_3_: Calcium carbonate
FTU: Phytase units
G:F: Gain to feed ratio
InsP: Inositol phosphate
InsP_6_: *myo*-inositol 1,2,3,4,5,6-hexakis (dihydrogen phosphate)
OTU: Operative taxonomic units
P: Phosphorus
pc: Prececal
PCR: Polymerase-chain
PERMANOVA: Permutational multivariate analysis of variance
pp.: Percentage point
SIMPER: Similarity percentage analysis

## References

[1] Sommerfeld V, Schollenberger M, Kühn I, Rodehutscord M (2018). Interactive effects of phosphorus, calcium, and phytase supplements on products of phytate degradation in the digestive tract of broiler chickens. Poult Sci.

[2] Selle PH, Cowieson AJ, Ravindran V (2009). Consequences of calcium interactions with phytate and phytase for poultry and pigs. Livest Sci.

[3] Plumstead PW, Leytem AB, Maguire RO, Spears JW, Kwanyuen P, Brake J (2008). Interaction of calcium and phytate in broiler diets. 1. Effects on apparent prececal digestibility and retention of phosphorus. Poult Sci.

[4] Hamdi M, Solà-Oriol D, Davin R, Perez JF (2015). Calcium sources and their interaction with the different levels of non-phytate phosphorus affect performance and bone mineralization in broiler chickens. Poult Sci.

[5] Xing R, Yang H, Wang X, Yu H, Liu S, Li P (2020). Effects of calcium source and calcium level on growth performance, immune organ indexes, serum components, intestinal microbiota, and intestinal morphology of broiler chickens. J Appl Poult Res.

[6] Angel R, Tamim NM, Applegate TJ, Dhandu AS, Ellestad LE (2002). Phytic acid chemistry: influence on phytin-phosphorus availability and phytase efficacy. J Appl Poult Res.

[7] Li W, Angel R, Kim S-W, Jiménez-Moreno E, Proszkowiec-Weglarz M, Plumstead PW (2018). Impacts of age and calcium on phytase efficacy in broiler chickens. Anim Feed Sci Tech.

[8] Lawlor PG, Lynch PB, Caffrey PJ, O’Reilly JJ, O’Connell MK (2005). Measurements of the acid-binding capacity of ingredients used in pig diets. Ir Vet J.

[9] Grynspan F, Cheryan M (1983). Calcium phytate: effect of pH and molar ratio on in vitro solubility. J Am Oil Chem Soc.

[10] Kim JW, Kim JH, Kil DY (2015). Dietary organic acids for broiler chickens. A review. Rev Colomb Cienc Pecu.

[11] van Immerseel F, Russell JB, Flythe MD, Gantois I, Timbermont L, Pasmans F (2006). The use of organic acids to combat Salmonella in poultry. A mechanistic explanation of the efficacy. Avian Pathol.

[12] Theron MM, Lues JR (2019). Organic acids and food preservation.

[13] Dibner JJ, Buttin P (2002). Use of organic acids as a model to study the impact of gut microflora on nutrition and metabolism. J Appl Poult Res.

[14] Toplaghaltsyan A, Bazukyan I, Trchounian A (2017). The effects of different carbon sources on the antifungal activity by lactic acid bacteria. Curr Microbiol.

[15] van Immerseel F, Fievez V, de Buck J, Pasmans F, Martel A, Haesebrouck F, Ducatelle R (2004). Poult Sci.

[16] Bang J, Lee SY (2018). Assimilation of formic acid and CO_2_ by engineered Escherichia coli equipped with reconstructed one-carbon assimilation pathways. Proc Natl Acad Sci U S A.

[17] Neveling DP, Ahire JJ, Laubscher W, Rautenbach M, Dicks LMT (2020). Genetic and phenotypic characteristics of a multi-strain probiotic for broilers. Curr Microbiol.

[18] Hayek SA, Shahbazi A, Worku M, Ibrahim SA (2013). Enzymatic activity of Lactobacillus reuteri grown in a sweet potato based medium with the addition of metal ions. Springerplus..

[19] Davoren MJ, Liu J, Castellanos J, Rodríguez-Malavé NI, Schiestl RH (2019). A novel probiotic, Lactobacillus johnsonii 456, resists acid and can persist in the human gut beyond the initial ingestion period. Gut Microbes.

[20] Wang H, Ni X, Qing X, Liu L, Xin J, Luo M (2018). Probiotic Lactobacillus johnsonii BS15 improves blood parameters related to immunity in broilers experimentally infected with subclinical necrotic enteritis. Front Microbiol.

[21] Kumar V, Yadav AN, Verma P, Sangwan P, Saxena A, Kumar K, Singh B (2017). β-Propeller phytases: diversity, catalytic attributes, current developments and potential biotechnological applications. Int J Biol Macromol.

[22] Shin S, Ha N-C, Oh B-C, Oh T-K, Oh B-H (2001). Enzyme mechanism and catalytic property of β propeller phytase. Structure..

[23] Burns DA, Ciurczak EW, editors. Handbook of near-infrared analysis. 3rd ed. Boca Raton: CRC Press; 2008.

[24] Kim S-W, Li W, Angel R, Plumstead PW (2019). Modification of a limestone solubility method and potential to correlate with in vivo limestone calcium digestibility. Poult Sci.

[25] Dersjant-Li Y, Evans C, Kumar A (2018). Effect of phytase dose and reduction in dietary calcium on performance, nutrient digestibility, bone ash and mineralization in broilers fed corn-soybean meal-based diets with reduced nutrient density. Anim Feed Sci Tech..

[26] Paiva DM, Walk CL, McElroy AP (2013). Influence of dietary calcium level, calcium source, and phytase on bird performance and mineral digestibility during a natural necrotic enteritis episode. Poult Sci.

[27] Bradbury EJ, Wilkinson SJ, Cronin GM, Thomson P, Walk CL, Cowieson AJ (2017). Evaluation of the effect of a highly soluble calcium source in broiler diets supplemented with phytase on performance, nutrient digestibility, foot ash, mobility and leg weakness. Anim Prod Sci.

[28] Bradbury EJ, Wilkinson SJ, Cronin GM, Walk CL, Cowieson AJ (2018). Effects of phytase, calcium source, calcium concentration and particle size on broiler performance, nutrient digestibility and skeletal integrity. Anim Prod Sci.

[29] Wise A, Gilburt DJ (1981). Binding of cadmium and lead to the calcium-phytate complex in vitro. Toxicol Lett.

[30] Jongbloed AW, Mroz Z, van der Weij-Jongbloed R, Kemme PA (2000). The effects of microbial phytase, organic acids and their interaction in diets for growing pigs. Livest Prod Sci.

[31] Proszkowiec-Weglarz M, Angel R (2013). Calcium and phosphorus metabolism in broilers: effect of homeostatic mechanism on calcium and phosphorus digestibility. J Appl Poult Res.

[32] Guinotte F, Nys Y, de Monredon F (1991). The effects of particle size and origin of calcium carbonate on performance and ossification characteristics in broiler chicks. Poult Sci.

[33] Gesellschaft für Ernährungsphysiologie. Empfehlungen zur Energie- und Nährstoffversorgung der Legehennen und Masthühner (Broiler). Frankfurt am Main: DLG-Verlag; 1999.

[34] Rodehutscord M, Dieckmann A, Witzig M, Shastak Y (2012). A note on sampling digesta from the ileum of broilers in phosphorus digestibility studies. Poult Sci.

[35] Kaewtapee C, Eklund M, Wiltafsky M, Piepho H-P, Mosenthin R, Rosenfelder P (2017). Influence of wet heating and autoclaving on chemical composition and standardized ileal crude protein and amino acid digestibility in full-fat soybeans for pigs. J Anim Sci.

[36] Camarinha-Silva A, Jáuregui R, Chaves-Moreno D, Oxley APA, Schaumburg F, Becker K (2014). Comparing the anterior nare bacterial community of two discrete human populations using Illumina amplicon sequencing. Environ Microbiol.

[37] Kozich JJ, Westcott SL, Baxter NT, Highlander SK, Schloss PD (2013). Development of a dual-index sequencing strategy and curation pipeline for analyzing amplicon sequence data on the MiSeq Illumina sequencing platform. Appl Environ Microbiol.

[38] Edgar RC, Haas BJ, Clemente JC, Quince C, Knight R (2011). UCHIME improves sensitivity and speed of chimera detection. Bioinformatics..

[39] Wang Q, Garrity GM, Tiedje JM, Cole JR (2007). Naive Bayesian classifier for rapid assignment of rRNA sequences into the new bacterial taxonomy. Appl Environ Microbiol.

[40] Wemheuer F, Taylor JA, Daniel R, Johnston E, Meinicke P, Thomas T, Wemheuer B (2018). Tax4Fun2. A R-based tool for the rapid prediction of habitat-specific functional profiles and functional redundancy based on 16S rRNA gene marker gene sequences.

[41] Yilmaz P, Parfrey LW, Yarza P, Gerken J, Pruesse E, Quast C (2014). The SILVA and “all-species living tree project (LTP)” taxonomic frameworks. Nucleic Acids Res.

[42] Kanehisa M, Sato Y, Kawashima M, Furumichi M, Tanabe M (2016). KEGG as a reference resource for gene and protein annotation. Nucleic Acids Res.

[43] McDonald D, Clemente JC, Kuczynski J, Rideout JR, Stombaugh J, Wendel D (2012). The biological observation matrix (BIOM) format or how I learned to stop worrying and love the ome-ome. Gigascience..

[44] Verband Deutscher Landwirtschaftlicher Untersuchungs- und Forschungsanstalten. Handbuch der Landwirtschaftlichen Versuchs- und Untersuchungsmethodik (VDLUFA-Methodenbuch), Vol. III. Die chemische Untersuchung von Futtermitteln. Darmstadt, Germany: VDLUFA-Verlag; 2007.

[45] Zeller E, Schollenberger M, Kühn I, Rodehutscord M (2015). Hydrolysis of phytate and formation of inositol phosphate isomers without or with supplemented phytases in different segments of the digestive tract of broilers. J Nutr Sci.

[46] Sommerfeld V, Künzel S, Schollenberger M, Kühn I, Rodehutscord M (2018). Influence of phytase or myo-inositol supplements on performance and phytate degradation products in the crop, ileum, and blood of broiler chickens. Poult Sci.

[47] ISO EN 30024. Animal feeding stuffs — Determination of phytase activity. 2009. https://www.iso.org/standard/45787.html. Accessed 06/2020.

[48] Siegert W, Hofmann P, Rodehutscord M. Effect of low-temperature drying on the nitrogenous compounds and inositol phosphates in broiler chicken and cecectomized laying hen excreta. Anim Sci J. 2021;92:e13484. 10.1111/asj.13484. 10.1111/asj.13484 PMC12874500 33398904

[49] Grubješić G, Titze N, Krieg J, Rodehutscord M (2019). Determination of in situ ruminal crude protein and starch degradation values of compound feeds from single feeds. Arch Anim Nutr.

[50] Siegert W, Ganzer C, Kluth H, Rodehutscord M (2018). Effect of particle size distribution of maize and soybean meal on the precaecal amino acid digestibility in broiler chickens. Br Poult Sci.

[51] Borda-Molina D, Zuber T, Siegert W (2019). Camarinha da Silva a, Feuerstein D, Rodehutscord M. effects of protease and phytase supplements on small intestinal microbiota and amino acid digestibility in broiler chickens. Poult Sci.

[52] Clarke KR, Warwick RM (2001). Change in marine communities: an approach to statistical analyses and interpretation.

