# Supplementary material for: Effects of calcium level and source, formic acid, and phytase on phytate degradation and the microbiota in the digestive tract of broiler chickens
Source: Anim Microbiome. 2021 Mar 15;3:23. doi: 10.1186/s42523-021-00083-7 (PMC7962351; doi:10.1186/s42523-021-00083-7)
Supplement: Supplementary file 1 — Additional file 1: Table S1. Growth performance. Table S2. Crop content and ileum digesta pH. Table S3. PERMANOVA of the microbial community in crop content and ileum digesta. Table S4. Relative abundance of OTUs in the crop content. Table S5. Relative abundance of OTUs in the ileum digesta. Table S6. Correlation between OTUs in the crop content and other measured traits. Table S7. Correlation between OTUs in the ileum content and other measured traits. Table S8. Influences on genes assigned to P-related pathways in crop content and ileum digesta. Table S9. Influences on genes assigned to enzymes related to inositol phosphate and myo-inositol degradation in crop content and ileum digesta. Table S10. InsP6 disappearance and prececal P digestibility. Table S11. InsP6, lower inositol phosphate isomers, and myo-inositol in the crop content. Table S12. InsP6, lower inositol phosphate isomers, and myo-inositol in the gizzard digesta. Table S13. InsP6, lower inositol phosphate isomers, and myo-inositol in the ileum digesta. Table S14. Composition of the experimental diets. Table S15. Analyses of experimental diets. Figure S1. Cluster analysis similarity in crop content and ileum digesta. Figure S2. Relationship crop pH and relative abundance of OTU2 in the crop content. Figure S3. Relation between the relative abundances of OTU1 and OTU2 with other measured traits. [file 42523_2021_83_MOESM1_ESM.pdf]

Effects of calcium level and source, acidification, and phytase on phytate degradation and the microbiota in the digestive tract of broiler chickens

Supplementary data

Table of contents

Table S1. Growth performance 2

Table S2. Crop content and ileum digesta pH 3

Table S3. PERMANOVA of the microbial community in crop content and ileum digesta 4

Table S4. Correlation between OTUs in the crop content and other measured traits 5

Table S5. Correlation between OTUs in the ileum content and other measured traits 6

Table S6. Influences on genes assigned to P-related pathways in crop content and ileum digesta 7

Table S7. Influences on genes assigned to enzymes related to inositol phosphate and *myo*-inositol degradation in crop content and ileum digesta 9

Table S8. $InsP_6$ disappearance and prececal P digestibility 10

Table S9. $InsP_6$, lower inositol phosphate isomers, and *myo*-inositol in the crop content 12

Table S10. $InsP_6$, lower inositol phosphate isomers, and *myo*-inositol in the gizzard digesta 14

Table S11. $InsP_6$, lower inositol phosphate isomers, and *myo*-inositol in the ileum digesta 16

Table S12. Relative abundance of OTUs in the crop content 19

Table S13. Relative abundance of OTUs in the ileum digesta 21

Table S14. Composition of the experimental diets 22

Table S15. Analyses of experimental diets 23

Figure S1. Cluster analysis similarity in crop content and ileum digesta 24

Figure S2. Relationship crop pH and relative abundance of OTU2 in the crop content 25

Figure S3. Relationship between the relative abundances of OTU1 and OTU2 with other measured traits 26

Table S1. Average daily gain (ADG), average daily feed intake (ADFI) and the gain to feed ratio (G:F) of broiler chickens fed with differently acidified diets with different Ca levels, and without (-) or with (+) supplementation of 1,500 FTU phytase/kg.

Acidification	Ca level	Phytase	ADG (g/d)	ADFI (g/d)	G:F (g/g)
Treatments					
$CaCO_3$	low	-	68.3	100.6	0.68
		+	74.1	99.9	0.74
	high	-	64.4	93.5	0.69
		+	73.2	99.7	0.74
$CaCO_3$+formic acid	low	-	71.3	98.4	0.72
		+	71.4	99.6	0.71
	high	-	62.5	92.1	0.68
		+	73.7	99.8	0.74
Ca-formate	low	-	72.0	99.4	0.72
		+	72.2	96.4	0.75
	high	-	58.8	88.0	0.67
		+	70.8	97.5	0.73
		SEM	2.13	1.31	0.019
Significant 2-way interactions[1]					
Ca level × Phytase	low	-	70.6^{a}	99.5^{a}	.
		+	72.6^{a}	98.7^{a}	
	high	-	61.9^{b}	91.2^{b}	.
		+	72.6^{a}	99.0^{a}	.
		SEM	1.36	0.87	
Significant main effects[2]					
$CaCO_3$			.	98.4^{a}	.
$CaCO_3$+formic acid			.	97.5^{a}	.
Ca-formate			.	95.3^{b}	.
SEM				0.80	
	low		.	.	.
	high		.	.	.
	SEM				
		-	.	.	0.69^{b}
		+	.	.	0.73^{a}
		SEM			0.008
ANOVA					
Acidification			0.520	0.002	0.934
Ca level			<0.001	<0.001	0.129
Phytase			<0.001	<0.001	<0.001
Acidification × Ca level			0.198	0.427	0.367
Acidification × Phytase			0.835	0.568	0.563
Ca level × Phytase			<0.001	<0.001	0.201
Acidification × Ca level × Phytase			0.245	0.155	0.334

[a,b] Values in the same column within a statistical comparison not sharing the same superscript letter are significantly different ($P \leq 0.050$)

[1] Presented if a 2-way interaction was significant ($P \leq 0.050$) and the 3-way interaction was not significant ($P > 0.050$).

[2] Presented if the main effect was significant ($P \leq 0.050$), and the 3-way interaction and the 2-way interactions were not significant ($P > 0.050$).

Table S2. Digesta pH in the digestive tract of broiler chickens fed with differently acidified diets with different Ca levels, and without (-) or with (+) supplementation of 1,500 FTU phytase/kg.

Acidification	Ca level	Phytase	Crop	Gizzard	Ileum
Treatments					
$CaCO_3$	low	-	5.5	2.8	7.0
		+	5.5	3.0	7.1
	high	-	5.4	3.3	6.6
		+	5.4	3.2	7.5
$CaCO_3$+formic acid	low	-	5.0	2.8	7.2
		+	4.9	3.0	7.5
	high	-	4.9	3.3	6.6
		+	4.9	3.2	7.7
Ca-formate	low	-	5.2	2.9	7.0
		+	5.2	2.9	7.3
	high	-	5.0	3.3	7.2
		+	5.2	3.1	7.7
		SEM	0.05	0.06	0.18
Significant 2-way interactions[1]					
Ca level × Phytase	low	-	.	2.8^{d}	7.1^{b}
		+	.	3.0^{c}	7.3^{b}
	high	-	.	3.3^{a}	6.8^{c}
		+	.	3.2^{b}	7.6^{a}
		SEM		0.04	0.12
Significant main effects[2]					
$CaCO_3$			5.5^{a}	.	.
$CaCO_3$+formic acid			5.0^{c}	.	.
Ca-formate			5.2^{b}	.	.
SEM			0.03		
	low		5.2^{a}	.	.
	high		5.1^{b}	.	.
	SEM		0.02		
ANOVA					
Acidification			<0.001	0.411	0.082
Ca level			0.002	<0.001	0.893
Phytase			0.560	0.644	<0.001
Acidification × Ca level			0.398	0.643	0.129
Acidification × Phytase			0.123	0.624	0.319
Ca level × Phytase			0.399	<0.001	<0.001
Acidification × Ca level × Phytase			0.887	0.671	0.257

[a-d] Values in the same column within a statistical comparison not sharing the same superscript letter are significantly different ($P \leq 0.050$)

[1] Presented if a 2-way interaction was significant ($P \leq 0.050$) and the 3-way interaction was not significant ($P > 0.050$).

[2] Presented if the main effect was significant ($P \leq 0.050$), and the 3-way interaction and the 2-way interactions were not significant ($P > 0.050$).

Table S3. PERMANOVA results (*P* values) of the microbial community in the crop content and ileum digesta of broiler chickens fed with differently acidified diets with different Ca levels without and with supplementation of 1,500 FTU phytase/kg.

	Crop	Ileum
Acidification	<0.001	0.012
Ca level	0.006	<0.001
Phytase	0.002	0.034
Acidification × Ca level	0.108	0.109
Acidification × Phytase	0.572	0.451
Ca level × Phytase	0.424	0.951
Acidification × Ca level × Phytase	0.628	0.151
Differences between acidification		
$CaCO_3$ vs. Ca-formate	<0.001	0.001
$CaCO_3$ vs. $CaCO_3$+formic acid	0.005	<0.001
Ca-formate vs. $CaCO_3$+formic acid	0.100	0.313

Table S4. Pearson correlation coefficients between the relative abundance of OTUs in the crop content of broiler chickens and other measured traits (n = 12 diets). Only significant (*P* ≤ 0.050) correlations are presented.

	crop pH	ADG	G:F	ADFI	$InsP_{3x}$[1,2]	Ins(1,2,3,4,6)P_5[2]	Ins(1,2,3,4,5)P_5[2]	Ins(1,2,4,5,6)P_5[2]	$InsP_6$[2]
OTU2	-0.31	-0.43	-0.34	-0.35					
OTU3		0.27		0.28					
OTU4	0.26	0.32		0.38		-0.24			
OTU5	0.25								
OTU6						0.30			
OTU7									
OTU8	-0.30			-0.28					
OTU9	0.45	0.33		0.38					
OTU10	0.25						-0.32		
OTU11	0.50	0.34		0.38		-0.25			
OTU12	-0.30								
OTU13	0.36	0.32		0.34					
OTU14				0.24	0.30				
OTU15	0.31								
OTU17					-0.26				
OTU18	-0.27								
OTU19	0.48								
OTU23	0.42								
OTU27	0.58								
OTU30								-0.24	-0.24

[1] At least one of the following inositol phosphate isomers: Ins(1,2,6)P_3, Ins(1,4,5)P_3, Ins(2,4,5)P_3.

[2] Concentrations in the crop.

Table S5. Pearson correlation coefficients between the relative abundance OTUs in the ileum digesta of broiler chickens and other measured traits (n = 12 diets). Only significant ($P \leq 0.050$) correlations are presented.

	crop pH	gizzard pH	ileum pH	preceal Ca digestibili-ty (%)	prececal P digestibili-ty (%)	prececal $InsP_6$ disappear-ance (%)	*myo*-inositol[2]	$InsP_{3x}$[1,2]	Ins(1,2,3,4,5)P_5[2]	Ins(1,2,4,5,6)P_5[2]	$InsP_6$[2]
OTU1	-0.29				0.35	0.34	0.44				-0.33
OTU2					-0.48	-0.47	-0.45			0.47	0.50
OTU4					0.29	0.30				-0.31	-0.32
OTU5	0.40										
OTU7			0.31								
OTU8	-0.32	0.36								0.46	
OTU9			-0.27	0.34	0.42	0.44	0.41			-0.28	-0.45
OTU10			-0.41						-0.27	-0.38	
OTU11			-0.30	0.38	0.39	0.40	0.41				-0.40
OTU13					0.29	0.31	0.26				-0.32
OTU17	0.26										
OTU23	0.28		-0.43						-0.28		
OTU27				-0.30				0.33			

[1] At least one of the following inositol phosphate isomers: Ins(1,2,6)P_3, Ins(1,4,5)P_3, Ins(2,4,5)P_3.
[2] Concentrations in the ileum.

Table S6. Relative proportion of genes assigned to P-related KEGG pathways in crop content and ileum digesta of broiler chickens fed with differently acidified diets with low and high Ca levels without (-) and with (+) supplementation of 1,500 FTU phytase/kg.

Acidification	Ca level	Phytase	InsP metabolism		Phosphotransferase system		Phosphatidylinositol signaling system		Phosphonate and phosphinate metabolism		Mineral absorption	
			Crop	Ileum	Crop	Ileum	Crop	Ileum	Crop	Ileum	Crop	Ileum
Treatments												
$CaCO_3$	low	-	0.163	0.130	1.60	1.96	0.067	0.054	0.035	0.025^{bc}	0.022	0.026
		+	0.157	0.124	1.68	1.95	0.066	0.055	0.034	0.029^{abc}	0.025	0.030
	high	-	0.144	0.117	1.75	1.93	0.061	0.052	0.034	0.033^{a}	0.028	0.033
		+	0.156	0.130	1.64	1.95	0.063	0.056	0.034	0.029^{abc}	0.025	0.029
$CaCO_3$+	low	-	0.134	0.119	1.85	2.05	0.057	0.050	0.029	0.025^{bc}	0.029	0.033
formic acid		+	0.148	0.120	1.77	2.09	0.060	0.054	0.029	0.025^{bc}	0.026	0.032
	high	-	0.127	0.122	1.87	1.90	0.056	0.053	0.032	0.031^{ab}	0.032	0.031
		+	0.133	0.123	1.86	1.99	0.060	0.054	0.031	0.027^{abc}	0.029	0.031
Ca-formate	low	-	0.144	0.114	1.73	2.02	0.059	0.052	0.032	0.029^{abc}	0.028	0.034
		+	0.145	0.118	1.80	2.11	0.059	0.051	0.029	0.023^{c}	0.027	0.032
	high	-	0.115	0.106	2.11	2.17	0.050	0.048	0.027	0.025^{bc}	0.037	0.037
		+	0.136	0.110	2.03	2.12	0.053	0.049	0.021	0.030^{abc}	0.031	0.035
	SEM		0.0086	0.0051	0.087	0.041	0.0020	0.0014	0.0018	0.0025	0.0021	0.0023
Significant 2-way interactions[1]												
Acidification × Ca level												
$CaCO_3$	low		.	.	.	1.95^{b}	0.067^{a}	0.054^{a}	0.034^{a}	.	.	.
	high		.	.	.	1.94^{b}	0.062^{b}	0.054^{a}	0.034^{a}	.	.	.
$CaCO_3$+	low		.	.	.	2.07^{a}	0.058^{c}	0.052^{a}	0.029^{b}	.	.	.
formic acid	high		.	.	.	1.95^{b}	0.058^{c}	0.053^{a}	0.031^{ab}	.	.	.
Ca-Formate	low		.	.	.	2.06^{a}	0.059^{bc}	0.052^{a}	0.031^{ab}	.	.	.
	high		.	.	.	2.15^{a}	0.061^{d}	0.049^{b}	0.024^{c}	.	.	.
SEM						0.029	0.0015	0.0011	0.0012			
Significant main effects[2]												
$CaCO_3$			0.155^{a}	0.125^{a}	1.67^{b}	.	.	.	.	.	0.025^{b}	0.030^{b}
$CaCO_3$+ formic acid			0.135^{b}	0.121^{a}	1.84^{a}	.	.	.	.	.	0.029^{a}	0.032^{ab}
Ca-formate			0.135^{b}	0.112^{b}	1.92^{a}	.	.	.	.	.	0.031^{a}	0.034^{a}
SEM			0.0050	0.0031	0.050						0.0012	0.0012
	low		0.149^{a}	.	1.74^{b}	.	.	.	.	.	0.026^{b}	.
	high		0.134^{b}	.	1.88^{a}	.	.	.	.	.	0.030^{a}	.
	SEM		0.0044								0.0010	

Table continued on next page.

Table S6. Continuation.

Acidification	Ca level	Phytase	InsP metabolism		Phosphotransferase system		Phosphatidylinositol signaling system		Phosphonate and phosphinate metabolism		Mineral absorption	
			Crop	Ileum	Crop	Ileum	Crop	Ileum	Crop	Ileum	Crop	Ileum
Significant main effects[2]												
		-	.	.	.	.	.	0.052[b]	0.032[a]	.	.	.
		+	.	.	.	.	.	0.053[a]	0.029[b]	.	.	.
		SEM						0.0008	0.007			
ANOVA												
Acidification			<0.001	0.002	<0.001	<0.001	<0.001	<0.001	<0.001	0.354	<0.001	0.022
Ca level			0.007	0.349	0.006	0.460	<0.001	0.305	0.120	0.047	0.002	0.258
Phytase			0.092	0.299	0.642	0.228	0.095	0.031	0.048	0.669	0.069	0.352
Acidification × Ca level			0.678	0.309	0.052	0.004	0.025	0.036	0.002	0.775	0.277	0.294
Acidification × Phytase			0.752	0.902	0.934	0.598	0.522	0.204	0.102	0.861	0.538	0.798
Ca level × Phytase			0.280	0.273	0.359	0.689	0.286	0.741	0.747	0.869	0.156	0.497
Acidification × Ca level × Phytase			0.397	0.266	0.479	0.240	0.981	0.154	0.687	0.029	0.477	0.335

[a-c] Values in the same column within a statistical comparison not sharing the same superscript letter are significantly different ($P \leq 0.050$)

[1] Presented if a 2-way interaction was significant ($P \leq 0.050$) and the 3-way interaction was not significant ($P > 0.050$).

[2] Presented if the main effect was significant ($P \leq 0.050$), and the 3-way interaction and the 2-way interactions were not significant ($P > 0.050$).

Table S7. Relative proportion of genes assigned to enzymes related to inositol phosphate and myo-inositol degradation listed in the KEGG database in crop content and ileum digesta of broiler chickens fed with differently acidified diets with low and high Ca levels without (-) and with (+) supplementation of 1,500 FTU phytase/kg.

Acidification	Ca level	Phytase	*myo*-inositol-1(or 4)-monophosphatase crop	*myo*-inositol-1(or 4)-monophosphatase ileum	*myo*-inositol-1-phosphatase synthase ileum
Treatments					
$CaCO_3$	low	-	0.035	0.016	1.3×10^{-4}
		+	0.034	0.017	6.1×10^{-5}
	high	-	0.027	0.013	7.3×10^{-5}
		+	0.030	0.019	1.2×10^{-4}
$CaCO_3$+	low	-	0.021	0.010	4.4×10^{-5}
formic acid		+	0.025	0.016	3.6×10^{-5}
	high	-	0.018	0.014	2.2×10^{-5}
		+	0.024	0.015	1.8×10^{-5}
Ca-formate	low	-	0.024	0.013	1.6×10^{-5}
		+	0.024	0.012	1.4×10^{-5}
	high	-	0.009	0.008	8.5×10^{-6}
		+	0.015	0.008	3.9×10^{-5}
	SEM		0.0030	0.0020	2.64×10^{-5}
Significant 2-way interactions[1]					
Acidification × Ca level					
$CaCO_3$	low		0.035[a]	0.017[a]	.
	high		0.028[b]	0.016[ab]	.
$CaCO_3$+	low		0.023[bc]	0.013[b]	.
formic acid	high		0.021[c]	0.015[ab]	.
Ca-Formate	low		0.024[bc]	0.013[b]	.
	high		0.012[d]	0.008[c]	.
SEM			0.0023	0.0015	
Significant main effects[2]					
$CaCO_3$			.	.	9.4×10^{-5} [a]
$CaCO_3$+ formic acid			.	.	3.0×10^{-5} [b]
Ca-formate			.	.	1.9×10^{-5} [b]
SEM					1.37×10^{-5}
ANOVA					
Acidification			<0.001	<0.001	<0.001
Ca level			<0.001	0.232	0.841
Phytase			0.055	0.040	0.937
Acidification × Ca level			0.039	0.027	0.726
Acidification × Phytase			0.571	0.164	0.796
Ca level × Phytase			0.208	0.747	0.121
Acidification × Ca level × Phytase			0.898	0.132	0.353

[a-c] Values in the same column within a statistical comparison not sharing the same superscript letter are significantly different ($P \leq 0.050$)

[1] Presented if a 2-way interaction was significant ($P \leq 0.050$) and the 3-way interaction was not significant ($P > 0.050$).

[2] Presented if the main effect was significant ($P \leq 0.050$), and the 3-way interaction and the 2-way interactions were not significant ($P > 0.050$).

Table S8. $InsP_6$ disappearance in the crop, prececal (pc) $InsP_6$ disappearance, pc P and Ca digestibility, and amount of pc digested P and Ca of broiler chickens fed with differently acidified diets with different Ca levels without (-) and with (+) supplementation of 1,500 FTU phytase/kg.

Acidification	Ca level	Phytase	$InsP_6$ disappearance in the crop (%)	pc $InsP_6$ disappearance (%)	pc P digestibility (%)	pc Ca digestibility (%)	pc digested P (g/d)	pc digested Ca (g/d)
Treatments								
$CaCO_3$	low	-	10	43	47	53^{d-f}	0.27^{b}	0.30^{f}
		+	27	83	74	61^{a}	0.31^{a}	0.34^{d}
	high	-	11	36	39	56^{c-e}	0.17^{c}	0.42^{b}
		+	36	83	68	55^{d-f}	0.16^{cd}	0.45^{ab}
$CaCO_3$+formic acid	low	-	15	42	47	53^{ef}	0.27^{b}	0.29^{f}
		+	50	84	76	62^{a}	0.31^{a}	0.34^{d}
	high	-	16	40	40	58^{bc}	0.17^{c}	0.44^{a}
		+	55	85	68	52^{f}	0.14^{de}	0.42^{bc}
Ca-formate	low	-	16	38	45	55^{c-f}	0.26^{b}	0.31^{f}
		+	16	77	71	61^{ab}	0.31^{a}	0.33^{d}
	high	-	9	20	32	54^{c-f}	0.13^{e}	0.39^{c}
		+	21	73	59	57^{cd}	0.17^{c}	0.46^{a}
		SEM	4.0	2.4	1.6	1.4	0.011	0.012
Significant 2-way interactions[1]								
Acidification × Ca level								
$CaCO_3$	low		.	63^{a}	60^{ab}	.	.	.
	high		.	59^{bc}	53^{c}	.	.	.
$CaCO_3$+formic acid	low		.	63^{ab}	61^{a}	.	.	.
	high		.	62^{ab}	54^{c}	.	.	.
Ca-formate	low		.	57^{c}	58^{b}	.	.	.
	high		.	46^{d}	45^{d}	.	.	.
	SEM			1.9	1.2			

Table continued on next page.

Table S8. Continuation.

Acidification	Ca level	Phytase	$InsP_6$ disappearance in the crop (%)	pc $InsP_6$ disappearance (%)	pc P digestibility (%)	pc Ca digestibility (%)	pc digested P (g/d)	pc digested Ca (g/d)
Significant 2-way interactions[1]								
Acidification × Phytase								
$CaCO_3$		-	11^{d}	.	.	.	.	.
		+	31^{b}	.	.	.	.	.
$CaCO_3$+formic acid		-	15^{cd}	.	.	.	.	.
		+	52^{a}	.	.	.	.	.
Ca-formate		-	13^{cd}	.	.	.	.	.
		+	19^{c}	.	.	.	.	.
		SEM	3.3					
Ca level × Phytase	low	-	14^{c}	41^{b}	.	.	.	.
		+	31^{b}	81^{a}	.	.	.	.
	high	-	12^{c}	32^{c}	.	.	.	.
		+	37^{a}	80^{a}	.	.	.	.
		SEM	3.0	1.8				
Significant main effects[2]								
		-	.	.	42^{b}	.	.	.
		+	.	.	69^{a}	.	.	.
		SEM			0.8			
ANOVA								
Acidification			<0.001	<0.001	<0.001	0.866	0.411	0.673
Ca level			0.239	<0.001	<0.001	0.004	<0.001	<0.001
Phytase			<0.001	<0.001	<0.001	<0.001	<0.001	<0.001
Acidification × Ca level			0.335	0.004	0.017	0.693	0.558	0.695
Acidification × Phytase			<0.001	0.687	0.739	0.300	0.082	0.265
Ca level × Phytase			0.043	0.002	0.451	<0.001	<0.001	0.232
Acidification × Ca level × Phytase			0.726	0.185	0.583	0.012	0.025	0.001

[a-d] Values in the same column within a statistical comparison not sharing the same superscript letter are significantly different ($P \leq 0.050$)

[1] Presented if a 2-way interaction was significant ($P \leq 0.050$) and the 3-way interaction was not significant ($P > 0.050$).

[2] Presented if the main effect was significant ($P \leq 0.050$), and the 3-way interaction and the 2-way interactions were not significant ($P > 0.050$).

Table S9. Concentration (µmol/g dry matter) of $InsP_6$, lower inositol phosphate isomers, and *myo*-inositol in the crop content of broiler chickens fed differently acidified diets with different Ca levels without (-) and with (+) supplementation of 1,500 FTU phytase/kg.

Acidification	Ca level	Phytase	*myo*-Inositol	$InsP_{3x}$[1]	Ins(1,2,3,4)P_4	Ins(1,2,5,6)P_4	Ins(1,2,3,4,6)P_5	Ins(1,2,3,4,5)P_5	Ins(1,2,4,5,6)P_5	$InsP_6$
Treatments										
$CaCO_3$	low	-	2.2	0.3	<LOQ	0.3	0.4	1.0	1.5	17.4
		+	2.2	1.6	1.2	1.0	0.3	0.8	1.1	14.8
	high	-	2.2	0.2	<LOQ	<LOQ	0.4	0.9	1.5	17.3
		+	2.2	1.6	1.0	0.9	0.2	0.6	1.0	13.1
$CaCO_3$+	low	-	2.2	<LOQ[2]	<LOQ	<LOQ	0.4	0.9	1.5	16.7
formic acid		+	2.2	3.0	1.9	1.3	<LOQ	0.5	0.7	10.0
	high	-	2.1	0.2	0.2	0.3	0.4	1.0	1.5	17.2
		+	2.2	3.4	1.5	1.1	0.2	0.5	0.6	9.0
Ca-formate	low	-	2.1	<LOQ	<LOQ	<LOQ	0.4	0.9	1.5	16.6
		+	2.2	0.5	0.4	0.4	0.3	0.8	1.3	16.0
	high	-	2.0	0.2	<LOQ	0.2	0.4	0.9	1.5	16.7
		+	2.1	0.4	0.2	0.2	0.3	0.8	1.4	16.0
	SEM		0.06	0.19	0.09	0.05	0.02	0.05	0.06	0.78
Significant 2-way interactions[3]										
Acidification × Phytase			.	.	.	.	.			
$CaCO_3$		-	.	.	.	.	.	0.9^{a}	1.5^{a}	17.4^{a}
		+	.	.	.	.	.	0.7^{c}	1.0^{c}	13.9^{c}
$CaCO_3$+formic acid		-	.	.	.	.	.	1.0^{a}	1.5^{a}	17.0^{ab}
		+	.	.	.	.	.	0.5^{d}	0.7^{d}	9.5^{d}
Ca-formate		-	.	.	.	.	.	0.9^{ab}	1.5^{a}	16.6^{ab}
		+	.	.	.	.	.	0.8^{bc}	1.3^{b}	16.0^{b}
		SEM						0.04	0.05	0.62

Table continued on next page.

Table S9. Continuation.

Acidification	Ca level	Phytase	*myo*-Inositol	$InsP_{3x}$[1]	Ins(1,2,3,4) P[4]	Ins(1,2,5,6) P_4	Ins(1,2,3, 4,6)P_5	Ins(1,2,3, 4,5)P_5	Ins(1,2,4, 5,6)P_5	$InsP_6$
ANOVA										
Acidification			0.069	<0.001	<0.001	<0.001	<0.001	0.013	<0.001	<0.001
Ca level			0.138	0.865	0.003	<0.001	0.311	0.092	0.866	0.322
Phytase			0.138	<0.001	<0.001	<0.001	<0.001	<0.001	<0.001	<0.001
Acidification × Ca level			0.643	0.347	0.382	0.203	0.911	0.194	0.570	0.620
Acidification × Phytase			0.643	<0.001	.	<0.001	0.004	<0.001	<0.001	<0.001
Ca level × Phytase			0.514	0.830	.	.	0.944	0.185	0.736	0.174
Acidification × Ca level × Phytase			0.859	.	.	.	0.668	0.845	0.331	0.759

[a-d] Values in the same column within a statistical comparison not sharing the same superscript letter are significantly different ($P \leq 0.050$)

[1] At least one of the following inositol phosphate isomers: Ins(1,2,6)P_3, Ins(1,4,5)P_3, Ins(2,4,5)P_3.

[2] <LOQ=below the limit of quantification in the majority of samples.

[3] Presented if quantifiable values were determined for each treatment, a 2-way interaction was significant ($P \leq 0.050$), and the 3-way interaction was not significant ($P > 0.050$).

Table S10. Concentration (µmol/g dry matter) of $InsP_6$, lower inositol phosphate isomers, and *myo*-inositol in the gizzard digesta of broiler chickens fed with differently acidified diets with different Ca levels without (-) and with (+) supplementation of 1,500 FTU phytase/kg.

Acidification	Ca level	Phytase	*myo*-Inositol	$InsP_{3x}$[1]	Ins(1,2,3,4)P_4	Ins(1,2,5,6)P_4	Ins(1,2,3,4,6)P_5	Ins(1,2,3,4,5)P_5	Ins(1,2,4,5,6)P_5	$InsP_6$
Treatments										
$CaCO_3$	low	-	1.6	0.1	nd	nd	0.2	0.4	0.5	8.7[b]
		+	2.5	2.6	0.2	0.2	nd	nd	nd	0.6[d]
	high	-	1.2	nd[2]	nd	nd	<LOQ	0.4	0.6	8.3[bc]
		+	1.9	2.5	<LOQ[3]	0.2	nd	nd	<LOQ	0.5[d]
$CaCO_3$+ formic acid	low	-	1.5	nd	nd	nd	<LOQ	0.4	0.5	8.0[c]
		+	2.8	2.5	nd	<LOQ	nd	nd	nd	0.5[d]
	high	-	1.2	nd	nd	nd	<LOQ	0.4	0.5	8.0[c]
		+	2.2	1.3	nd	nd	nd	nd	nd	0.4[d]
Ca-formate	low	-	1.5	nd	nd	nd	<LOQ	0.4	0.6	9.2[a]
		+	2.8	2.7	0.2	0.2	nd	nd	nd	0.6[d]
	high	-	1.1	nd	nd	nd	<LOQ	0.4	0.5	8.0[c]
		+	2.0	2.6	0.3	0.2	nd	nd	nd	0.6[d]
		SEM	0.11	0.24	0.10	0.07	.	0.03	0.03	0.18
Significant 2-way interactions[4]										
Ca level × Phytase										
	low	-	1.5[c]	.	.	.	.	.	.	.
		+	2.7[a]	.	.	.	.	.	.	.
	high	-	1.2[d]	.	.	.	.	.	.	.
		+	2.1[b]	.	.	.	.	.	.	.
		SEM	0.07							
ANOVA										
Acidification			0.277	0.012	0.854	1.000	.	0.239	0.344	0.007
Ca level			<.0001	0.018	0.854	0.867	.	0.059	0.633	0.003
Phytase			<.0001	<0.001	.	.	.	.	.	<0.001
Acidification × Ca level			0.611	0.051	.	.	.	0.369	0.250	0.049
Acidification × Phytase			0.058	.	.	.	.	.	.	0.290
Ca level × Phytase			0.022	.	.	.	.	.	.	0.026
Acidification × Ca level × Phytase			0.806	.	.	.	.	.	.	0.019

Footnotes to table on next page.

Footnotes to Table S10:

[a-d] Values in the same column within a statistical comparison not sharing the same superscript letter are significantly different ($P \leq 0.050$).

[1] At least one of the following inositol phosphate isomers: Ins(1,2,6)P_3, Ins(1,4,5)P_3, Ins(2,4,5)P_3.

[2] nd=below the detection limit in the majority of samples.

[3] <LOQ=below the limit of quantification in the majority of samples.

[4] Presented if quantifiable values were determined for each treatment, a 2-way interaction was significant ($P \leq 0.050$), and the 3-way interaction was not significant ($P > 0.050$).

Table S11. Concentration (µmol/g dry matter) of $InsP_6$, lower inositol phosphate isomers, and *myo*-inositol in the ileum digesta of broiler chickens fed with differently acidified diets with different Ca levels without (-) and with (+) supplementation of 1,500 FTU phytase/kg.

Acidification	Ca level	Phytase	*myo*-Inositol	$InsP_{3x}$[1]	InsP(1,5,6)P_3	Ins(1,2,3,4)P_4	Ins(1,2,5,6)P_4	Ins(1,2,3,4,6)P_5	Ins(1,2,3,4,5)P_5	Ins(1,2,4,5,6)P_5	$InsP_6$
Treatments											
$CaCO_3$	low	-	11.2	0.5	0.2	0.4^{c}	nd	0.6	1.0^{f}	0.5	27.2
		+	23.8	1.0	<LOQ[2]	2.1^{b}	0.8	<LOQ	1.8^{de}	0.5	8.5
	high	-	8.1	0.7	nd[3]	0.8^{c}	0.2	0.7	1.4^{def}	0.9	31.4
		+	18.5	1.9	<LOQ	2.9^{b}	1.1	<LOQ	1.9^{cd}	0.5	9.0
$CaCO_3$+	low	-	11.8	0.4	0.3	0.4^{c}	0.2	0.6	1.1^{f}	0.6	29.5
formic acid		+	28.3	1.1	nd	2.2^{b}	0.9	<LOQ	1.9^{cde}	0.5	9.0
	high	-	8.9	0.7	0.5	0.7^{c}	0.2	0.7	1.3^{ed}	0.9	31.4
		+	20.0	3.2	0.2	5.1^{a}	2.8	0.1	2.8^{b}	0.9	8.4
Ca-formate	low	-	11.2	0.5	nd	0.4^{c}	0.1	0.7	1.1^{f}	0.6	31.9
		+	24.3	0.9	<LOQ	2.5^{b}	1.0	<LOQ	2.4^{bc}	0.7	12.0
	high	-	6.8	0.5	0.3	0.3^{c}	0.2	0.7	1.4^{edf}	1.3	37.6
		+	15.8	1.6	0.2	5.0^{a}	2.4	0.2	4.1^{a}	1.4	14.3
	SEM		0.83	0.22	0.12	0.34	0.18	0.03	0.22	0.10	1.46
Significant 2-way interactions[4]											
Acidification × Ca level											
$CaCO_3$	low		.	0.8^{c}	.	.	.	.	.	0.5^{d}	.
	high		.	1.3^{b}	.	.	.	.	.	0.7^{c}	.
$CaCO_3$+	low		.	0.8^{c}	.	.	.	.	.	0.6^{cd}	.
formic acid	high		.	1.9^{a}	.	.	.	.	.	0.9^{b}	.
Ca-formate	low		.	0.7^{c}	.	.	.	.	.	0.6^{cd}	.
	high		.	1.0^{bc}	.	.	.	.	.	1.3^{a}	.
	SEM			0.16						0.08	

Table continued on next page.

Table S11. Continuation.

Acidification	Ca level	Phytase	*myo*-Inositol	$InsP_{3x}$[1]	InsP(1,5,6)P_3	Ins(1,2,3,4)P_4	Ins(1,2,5,6)P_4	Ins(1,2,3,4,6)P_5	Ins(1,2,3,4,5)P_5	Ins(1,2,4,5,6)P_5	$InsP_6$
Significant 2-way interactions[4]											
Acidification × Phytase											
$CaCO_3$		-	.	0.6^{c}	.	.	.	.	.	.	.
		+	.	1.4^{b}	.	.	.	.	.	.	.
$CaCO_3$+formic acid			.	0.6^{c}	.	.	.	.	.	.	.
		+	.	2.2^{a}	.	.	.	.	.	.	.
Ca-formate		-	.	0.5^{c}	.	.	.	.	.	.	.
		+	.	1.2^{b}	.	.	.	.	.	.	.
		SEM		0.16							
Ca level × Phytase											
	low	-	11.4^{c}	0.5^{c}	.	.	.	.	.	.	29.6^{b}
		+	25.5^{a}	1.0^{b}	.	.	.	.	.	.	9.8^{c}
	high	-	7.9^{d}	0.6^{c}	.	.	.	.	.	.	33.5^{a}
		+	18.1^{b}	2.2^{a}	.	.	.	.	.	.	10.6^{c}
		SEM	0.50	0.14	.	.	.	.	.	.	1.14
Significant main effects[5]											
$CaCO_3$			15.4^{b}	.	.	.	.	.	.	.	19.0^{b}
$CaCO_3$+formic acid			17.3^{a}	.	.	.	.	.	.	.	19.6^{b}
Ca-formate			14.5^{b}	.	.	.	.	.	.	.	24.0^{a}
SEM			0.43								1.10

Table continued on next page.

Table S11. Continuation.

Acidification	Ca level	Phytase	*myo*-Inositol	$InsP_{3x}$[1]	InsP(1,5,6)P_3	Ins(1,2,3,4)P_4	Ins(1,2,5,6)P_4	Ins(1,2,3,4,6)P_5	Ins(1,2,3,4,5)P_5	Ins(1,2,4,5,6)P_5	$InsP_6$
ANOVA											
Acidification			<0.001	0.006	0.635	0.040	0.021	0.007	<0.001	<0.001	<0.001
Ca level			<0.001	<0.001	0.198	<0.001	<0.001	<0.001	<0.001	<0.001	<0.001
Phytase			<0.001	<0.001	0.084	<0.001	<0.001	<0.001	<0.001	0.166	<0.001
Acidification × Ca level			0.159	0.023	.	0.080	<0.001	0.855	0.046	<0.001	0.112
Acidification × Phytase			0.059	0.010	0.718	<0.001	<0.001	0.965	<0.001	0.242	0.715
Ca level × Phytase			<0.001	<0.001	.	<0.001	<0.001	.	0.012	0.288	0.020
Acidification × Ca level × Phytase			0.414	0.081	.	0.030	0.239	.	0.023	0.276	0.938

[a-f] Values in the same column within a statistical comparison not sharing the same superscript letter are significantly different ($P \leq 0.050$)

[1] At least one of the following inositol phosphate isomers: Ins(1,2,6)P_3, Ins(1,4,5)P_3, Ins(2,4,5)P_3.

[2] <LOQ=below the limit of quantification in the majority of samples.

[3] nd=below the detection limit in the majority of samples.

[4] Presented if quantifiable values were determined for each treatment, a 2-way interaction was significant ($P \leq 0.050$), and the 3-way interaction was not significant ($P > 0.050$).

[5] Presented if quantifiable values were determined for each treatment, the main effect was significant ($P \leq 0.050$), and the 3-way interaction and the 2-way interactions were not significant ($P > 0.050$).

Table S12. Relative abundance of OTUs in crop content of broiler chickens fed with differently acidified diets with different Ca levels without (-) and with (+) supplementation of 1,500 FTU phytase/kg. Only significantly influenced OTUs with a relative abundance >1% in at least one treatment are presented.

Acidification	Ca level	Phytase	OTU1	OTU2	OTU4	OTU5	OTU6	OTU8	OTU9	OTU10	OTU11	OTU12	OTU13	OTU14	OTU15	OTU16	OTU17	OTU19	OTU23
Treatments																			
$CaCO_3$	low	-	34.5	11.1	8.9	5.4	3.8	1.4	3.5	1.5	2.8	0.4	2.0	0.9^{b}	2.0	1.3^{abc}	0.8	1.6	1.2
		+	41.1	12.0	11.0	4.2	2.7	1.3	3.7	0.9	2.6	0.2	2.2	0.4^{b}	1.0	2.2^{abc}	0.6	1.0	0.7
	high	-	35.5	20.7	5.8	<0.1	4.6	1.6	2.4	0.2	1.6	0.4	1.1	1.8^{ab}	2.2	0.5^{bc}	2.2	1.6	0.5
		+	35.2	16.1	5.1	0.2	6.5	1.0	2.3	4.2	1.9	0.5	1.1	0.2^{b}	2.6	3.7^{abc}	0.5	1.9	1.1
$CaCO_3$+	low	-	38.5	18.3	5.1	<0.1	5.3	2.1	2.2	<0.1	1.6	1.8	1.2	1.1^{ab}	1.2	4.8^{a}	2.0	0.1	<0.1
formic acid		+	41.4	12.7	7.3	<0.1	3.1	6.3	2.8	<0.1	2.0	4.7	1.7	1.9^{ab}	0.3	1.1^{bc}	0.2	<0.1	<0.1
	high	-	33.8	28.4	5.0	<0.1	4.3	5.6	1.4	<0.1	1.0	2.7	0.8	2.2^{ab}	0.9	<0.1^{c}	1.8	0.2	0.1
		+	38.8	19.6	7.6	<0.1	3.5	3.3	2.2	<0.1	1.5	2.3	1.5	2.2^{ab}	1.0	3.8^{a}	0.3	<0.1	0.3
Ca-formate	low	-	35.6	22.8	6.5	<0.1	4.2	4.1	1.6	<0.1	1.1	3.2	0.9	3.0^{a}	0.7	1.2^{abc}	<0.1	0.1	0.1
		+	46.0	13.4	7.4	<0.1	3.3	2.8	3.9	<0.1	2.6	1.8	2.4	0.2^{b}	0.5	2.1^{abc}	<0.1	0.2	0.4
	high	-	41.3	28.7	2.0	<0.1	1.2	6.8	1.0	<0.1	0.6	4.0	0.6	0.4^{b}	0.2	1.5^{abc}	0.7	<0.1	0.4
		+	52.8	12.3	4.7	<0.1	1.2	6.9	1.6	<0.1	1.2	3.8	1.1	1.3^{ab}	0.1	0.2^{bc}	0.5	<0.1	0.3
	SEM		2.96	3.93	1.76	1.41	1.26	1.36	0.48	1.05	0.36	0.94	0.38	0.76	0.40	1.30	0.69	0.39	0.28
Significant 2-way interactions[1]																			
Acidification × Ca level													.		.			
$CaCO_3$	low		37.8^{b}	.	.	.	3.2^{bc}	.	.	.	.	.	.	.	.	.	.	.	.
	high		35.3^{b}	.	.	.	5.6^{a}	.	.	.	.	.	.	.	.	.	.	.	.
$CaCO_3$+	low		40.0^{b}	.	.	.	4.2^{ab}	.	.	.	.	.	.	.	.	.	.	.	.
formic acid	high		36.3^{b}	.	.	.	3.9^{ab}	.	.	.	.	.	.	.	.	.	.	.	.
Ca-Formate	low		40.8^{b}	.	.	.	3.7^{ab}	.	.	.	.	.	.	.	.	.	.	.	.
	high		47.1^{a}	.	.	.	1.2^{c}	.	.	.	.	.	.	.	.	.	.	.	.
SEM			2.06				1.01												
Acidification × Phytase																			
$CaCO_3$		-	.	.	.	4.8^{a}	.	.	.	.	.	.	.	.	.	.	.	.	.
		+	.	.	.	0.1^{b}	.	.	.	.	.	.	.	.	.	.	.	.	.
$CaCO_3$+		-	.	.	.	<0.1^{b}	.	.	.	.	.	.	.	.	.	.		.	.
formic acid		+	.	.	.	<0.1^{b}	.	.	.	.	.	.	.	.	.	.	.	.	.
Ca-formate		-	.	.	.	<0.1^{b}	.	.	.	.	.	.	.	.	.	.	.	.	.
		+	.	.	.	<0.1^{b}	.	.	.	.	.	.	.	.	.	.	.	.	.
		SEM				0.98													

Table continued on next page.

Table S12. Continuation.

Acidification	Ca level	Phytase	OTU1	OTU2	OTU4	OTU5	OTU6	OTU8	OTU9	OTU10	OTU11	OTU12	OTU13	OTU14	OTU15	OTU16	OTU17	OTU19	OTU23
Significant main effects[2]																			
$CaCO_3$			.	.	.	.	.	5.2[a]	3.0[a]	1.7[a]	2.2[a]	0.4[b]	.	.	1.9[a]	.	.	1.5[a]	0.9[a]
$CaCO_3$+ formic acid			.	.	.	.	.	4.4[a]	2.1[b]	<0.1[b]	1.5[b]	2.9[a]	.	.	0.8[b]	.	.	<0.1[b]	0.3[b]
Ca-formate			.	.	.	.	.	1.3[b]	2.0[b]	<0.1[b]	1.4[b]	3.2[a]	.	.	0.4[b]	.	.	<0.1[b]	0.1[b]
SEM								0.76	0.29	0.57	0.22	0.52			0.22			0.21	0.14
	low		.	15.0[b]	7.7[a]	.	.	.	2.9[a]	.	2.1[a]	.	1.8[a]	.	.	.	.	.	.
	high		.	21.0[a]	5.0[b]	.	.	.	1.8[b]	.	1.3[b]	.	1.0[b]	.	.	.	.	.	.
	SEM			2.04	1.01				0.26		0.20		0.22						
		-	36.5[b]	21.7[a]	.	.	.	.	2.0[b]	.	1.5[a]	.	1.1[b]	.	.	.	1.3[a]	.	.
		+	42.5[a]	14.3[b]	.	.	.	.	2.8[a]	.	2.0[b]	.	1.7[a]	.	.	.	0.4[b]	.	.
		SEM	1.12	2.04					0.26		0.20		0.22						
ANOVA																			
Acidification			0.003	0.142	0.083	0.025	0.032	<0.001	0.006	0.030	0.001	<0.001	0.267	0.147	<0.001	0.445	0.206	<0.001	0.001
Ca level			0.987	0.007	0.005	0.064	0.795	0.130	<0.001	0.607	<0.001	0.585	<0.001	0.216	0.295	0.902	0.312	0.713	0.895
Phytase			0.001	0.001	0.083	0.855	0.378	0.940	0.004	0.333	0.009	0.815	0.004	0.790	0.219	0.953	0.025	0.694	0.584
Acidification × Ca level			0.047	0.493	0.097	0.036	0.008	0.110	0.471	0.754	0.590	0.247	0.345	0.290	0.053	0.187	0.718	0.550	0.744
Acidification × Phytase			0.139	0.114	0.725	0.964	0.423	0.604	0.073	0.394	0.093	0.294	0.171	0.372	0.949	0.953	0.259	0.928	0.996
Ca level × Phytase			0.722	0.223	0.926	0.793	0.144	0.193	0.256	0.194	0.678	0.543	0.356	0.462	0.052	0.105	0.587	0.512	0.345
Acidification × Ca level × Phytase			0.522	0.929	0.569	0.928	0.773	0.077	0.310	0.188	0.288	0.172	0.409	0.041	0.428	0.037	0.575	0.557	0.191

[a-c] Values in the same column within a statistical comparison not sharing the same superscript letter are significantly different ($P \leq 0.050$).

[1] Presented if a 2-way interaction was significant ($P \leq 0.050$) and the 3-way interaction was not significant ($P > 0.050$).

[2] Presented if the main effect was significant ($P \leq 0.050$), and the 3-way interaction and the 2-way interactions were not significant ($P > 0.050$).

Table S13. Relative abundance of OTUs in ileum digesta of broiler chickens fed with differently acidified diets with different Ca levels without (-) and with (+) supplementation of 1,500 FTU phytase/kg. Only significantly influenced OTUs with a relative abundance >1% in at least one treatment are presented.

Acidification	Ca level	Phytase	OTU1	OTU2	OTU5	OTU6	OTU7	OTU8	OTU10	OTU15	OTU20
Treatments											
$CaCO_3$	low	-	25.3	16.5	13.3	0.9	5.9	0.1	6.9	0.4	0.3
		+	23.6	15.4	20.6	2.9	3.3	0.6	15.6	0.8	0.2
	high	-	26.9	40.0	0.4	3.1	1.7	0.9	5.5	0.9	0.5
		+	34.7	21.5	6.2	2.8	4.0	0.5	2.7	1.1	1.5
$CaCO_3$+	low	-	33.3	27.8	0.3	3.9	8.1	1.3	0.2	0.9	3.2
formic acid		+	45.6	19.7	0.2	2.9	4.3	1.7	0.1	0.4	1.6
	high	-	28.0	35.3	<0.1	4.1	2.3	2.2	4.9	0.7	0.3
		+	35.8	27.9	0.2	2.9	5.2	1.0	0.2	0.5	2.5
Ca-formate	low	-	35.1	35.8	0.1	1.0	4.5	0.7	0.2	0.2	3.0
		+	39.3	21.5	3.1	1.7	5.9	1.2	3.9	0.4	1.7
	high	-	40.6	35.9	0.2	0.6	3.4	2.2	0.3	0.1	0.5
		+	37.4	33.1	<0.1	0.4	5.9	1.6	0.2	<0.1	1.9
	SEM		3.71	6.13	3.85	1.13	1.52	0.37	3.67	0.28	1.02
Significant 2-way interactions[1]											
Acidification × Ca level											
$CaCO_3$	low		30.8^{cd}	.	17.0^{a}	.	.	.	.	.	.
	high		24.4^{d}	.	3.3^{b}	.	.	.	.	.	.
$CaCO_3$+form	low		31.9^{bc}	.	1.6^{b}	.	.	.	.	.	.
ic acid	high		39.4^{a}	.	0.2^{b}	.	.	.	.	.	.
Ca-Formate	low		39.0^{ab}	.	0.1^{b}	.	.	.	.	.	.
	high		37.2^{abc}	.	<0.1^{b}	.	.	.	.	.	.
SEM			2.73		2.71						
Ca level × Phytase											
	low	-	.	.	.	.	6.2^{a}	0.7^{b}	.	.	2.1^{a}
		+	.	.	.	.	4.5^{ab}	1.2^{b}	.	.	1.1^{ab}
	high	-	.	.	.	.	2.5^{b}	1.8^{a}	.	.	0.4^{b}
		+	.	.	.	.	5.0^{a}	1.0^{b}	.	.	1.9^{a}
		SEM					1.05	0.21			0.78
Significant main effects[2]											
$CaCO_3$			.	.	.	2.4^{a}	.	0.5^{b}	7.7^{a}	0.8^{a}	0.6^{b}
$CaCO_3$+ formic acid			.	.	.	3.5^{a}	.	1.6^{a}	1.4^{b}	0.6^{a}	1.9^{a}
Ca-formate			.	.	.	0.9^{b}	.	1.4^{b}	1.1^{b}	0.2^{b}	1.8^{ab}
SEM						0.78		0.19	1.96	0.13	0.74
	low		.	22.8^{b}	.	.	.	.	.	.	.
	high		.	32.3^{a}	.	.	.	.	.	.	.
	SEM			2.85							
		-	31.5^{b}	31.9^{a}	.	.	.	.	.	.	.
		+	36.1^{a}	23.2^{b}	.	.	.	.	.	.	.
		SEM	1.56	2.85							
ANOVA											
Acidification			<0.001	0.174	<0.001	0.003	0.329	<0.001	0.019	0.021	0.049
Ca level			0.928	0.009	0.029	0.852	0.053	0.031	0.306	0.781	0.340
Phytase			0.040	0.016	0.241	0.989	0.573	0.522	0.713	0.861	0.580
Acidification × Ca level			0.027	0.541	0.031	0.387	0.612	0.243	0.165	0.341	0.184
Acidification × Phytase			0.176	0.969	0.455	0.321	0.410	0.656	0.523	0.258	0.938
Ca level × Phytase			0.842	0.801	0.745	0.313	0.009	0.005	0.116	0.901	0.010
Acidification × Ca level × Phytase			0.232	0.240	0.953	0.689	0.337	0.834	0.702	0.866	0.458

[a,b] Values in the same column within a statistical comparison not sharing the same superscript letter are significantly different ($P \leq 0.050$).

[1] Presented if a 2-way interaction was significant ($P \leq 0.050$) and the 3-way interaction was not significant ($P > 0.050$).

[2] Presented if the main effect was significant ($P \leq 0.050$), and the 3-way interaction and the 2-way interactions were not significant ($P > 0.050$).

Table S14. Composition of the experimental diets (g/kg, unless otherwise stated).

<table>
<tr><td>Acidification</td><td colspan="4">$CaCO_3$</td><td colspan="4">$CaCO_3$+formic acid</td><td colspan="4">Ca-formate</td></tr>
<tr><td>Ca level</td><td colspan="2">low</td><td colspan="2">high</td><td colspan="2">low</td><td colspan="2">high</td><td colspan="2">low</td><td colspan="2">high</td></tr>
<tr><td>Phytase</td><td>-</td><td>+</td><td>-</td><td>+</td><td>-</td><td>+</td><td>-</td><td>+</td><td>-</td><td>+</td><td>-</td><td>+</td></tr>
<tr><td>$CaCO_3$</td><td colspan="2">7.32</td><td colspan="2">13.83</td><td colspan="2">7.32</td><td colspan="2">13.83</td><td colspan="2">0</td><td colspan="2">0</td></tr>
<tr><td>Formic acid</td><td colspan="2">0</td><td colspan="2">0</td><td colspan="2">6</td><td colspan="2">6</td><td colspan="2">0</td><td colspan="2">0</td></tr>
<tr><td>Ca-formate</td><td colspan="2">0</td><td colspan="2">0</td><td colspan="2">0</td><td colspan="2">0</td><td colspan="2">9.51</td><td colspan="2">18.2</td></tr>
<tr><td>Diamol</td><td colspan="2">12.51</td><td colspan="2">6</td><td colspan="2">6.51</td><td colspan="2">0</td><td colspan="2">10.32</td><td colspan="2">1.63</td></tr>
<tr><td>Phytase (FTU/kg)[1]</td><td>0</td><td>1,500</td><td>0</td><td>1,500</td><td>0</td><td>1,500</td><td>0</td><td>1,500</td><td>0</td><td>1,500</td><td>0</td><td>1,500</td></tr>
<tr><td>Corn</td><td colspan="12">440.67</td></tr>
<tr><td>Soybean meal</td><td colspan="12">300</td></tr>
<tr><td>Rapeseed meal</td><td colspan="12">100</td></tr>
<tr><td>Sunflower meal</td><td colspan="12">100</td></tr>
<tr><td>Soybean oil</td><td colspan="12">25</td></tr>
<tr><td>Premix[2]</td><td colspan="12">5</td></tr>
<tr><td>Titanium dioxide</td><td colspan="12">5</td></tr>
<tr><td>NaCl</td><td colspan="12">4</td></tr>
<tr><td>DL-Methionine</td><td colspan="12">0.5</td></tr>
</table>

[1] Added on top of the diets.

[2] Supplied per kg of diet: 12,000 IU vitamin A (retinyl acetate), 2,500 IU vitamin D3 (cholecalciferol), 50 mg vitamin E (DL-α-tocopherol), 1.5 mg vitamin K3 (menadione), 2.0 mg vitamin B1 (thiamine), 7.5 mg vitamin B2 (riboflavin), 3.5 mg vitamin B6 (pyridoxine), 20 µg vitamin B12 (cyanocobalamin), 30 mg niacin, 12 mg pantothenic acid, 460 mg choline chloride, 1.0 mg folic acid, 0.2 mg biotin, 80 mg iron, 12 mg copper, 85 mg manganese, 60 mg zinc, 0.8 mg iodine, 0.15 mg selenium, 125 mg anti-oxidant.

Table S15. Analyzed concentrations of nutrients, inositol phosphate isomers, phytase activity, and particle size distribution in the experimental diets (g/kg dry matter, unless otherwise stated).

Acidification	$CaCO_3$[1]				$CaCO_3$+formic acid				Ca-formate[1]			
Ca level	low		high		low		high		low		high	
Phytase[2]	-	+	-	+	-	+	-	+	-	+	-	+
Dry matter (g/kg)	884	885	888	888	886	886	886	885	888	887	891	888
Crude protein	266	261	263	262	264	262	262	262	259	263	259	262
Ether extract	62.5	58.4	60.1	58.3	57.8	57.1	58.2	57.8	56.4	56.7	56.8	55.9
Crude fiber	75.6	73.2	77.8	76.3	75.6	77.0	76.0	75.5	74.8	74.1	77.5	75.1
Crude ash	71.9	70.9	72.9	71.3	67.5	64.9	67.1	65.7	70.2	70.3	68.0	67.5
Calcium	5.6	5.6	8.0	8.1	5.5	5.5	8.2	8.1	5.7	5.6	8.3	8.2
Phosphorus	5.3	5.3	5.3	5.2	5.3	5.3	5.4	5.3	5.2	5.4	5.3	5.3
$InsP_6$-P	3.6	3.5	3.5	3.5	3.5	3.5	3.6	3.6	3.5	3.5	3.3	3.6
Gross energy (MJ/kg dry matter)	19.5	19.7	19.6	19.8	20.1	19.6	19.8	19.5	20.0	19.6	19.8	19.7
Inositol phosphates[3] and *myo*-inositol (µmol/g dry matter)												
$InsP_6$	19.2	18.8	18.8	19.0	18.8	19.3	18.7	19.1	18.6	17.8	18.7	19.2
Ins(1,2,4,5,6)P_5	1.6	1.7	1.7	1.7	1.6	1.6	1.6	1.7	1.6	1.6	1.7	1.6
Ins(1,2,3,4,5)P_5	1.0	1.0	1.0	1.0	1.0	0.9	1.0	1.0	0.9	0.9	1.0	0.9
Ins(1,2,3,4,6)P_5	0.4	0.5	0.5	0.4	0.4	0.4	0.4	0.5	0.5	0.4	0.4	0.4
Myo-inositol	2.1	2.1	2.1	2.1	2.1	2.1	2.1	2.1	2.1	2.1	2.1	2.1
Phytase (FTU/kg)	< 60	1,450	< 60	1,580	< 60	1,460	< 60	1,450	< 60	1,470	< 60	1,480
Particle size distribution[4]												
a[5]	2.06	2.19	1.95	1.91	2.11	2.35	2.06	1.92	2.17	2.29	2.46	2.23
	(0.05)	(0.06)	(0.10)	(0.12)	(0.06)	(0.08)	(0.10)	(0.07)	(0.08)	(0.08)	(0.21)	(0.07)
b[6] (mm)	0.290	0.181	0.264	0.182	0.287	0.280	0.226	0.271	0.188	0.130	0.202	0.231
	(0.008)	(0.011)	(0.014)	(0.022)	(0.010)	0.012)	(0.013)	(0.013)	(0.011)	(0.009)	(0.027)	(0.010)
R^2	0.995	0.991	0.986	0.968	0.993	0.990	0.986	0.989	0.991	0.993	0.945	0.993
Root mean square error (%)	1.54	1.99	2.54	3.66	1.89	2.22	2.83	2.29	2.03	1.87	5.01	1.95

[1] Estimated term coefficients of particle size distribution of Ca sources according to Equation 3: a = 0.215 (standard error (SE) 0.0062), b = 9.53 µm (SE 0.153), R^2 = 0.991, root mean square error = 3.6% for $CaCO_3$ and a = 0.0334 (SE 0.00094), b = 104 µm (SE 1.15), R^2 = 0.996, root mean square error = 2.9% for Ca-formate.

[2] - without phytase supplementation; + with supplementation of 1,500 FTU phytase/kg.

[3] Inositol phosphate isomers not shown were below the limit of quantification in all diets.

[4] Estimates according to Equation 3. SE of estimated term coefficients are shown in parentheses.

[5] Slope of the regression; the higher the estimates the more pronounced is heterogeneity of particle size distribution.

[6] Inflection point; indicates mean particle size.

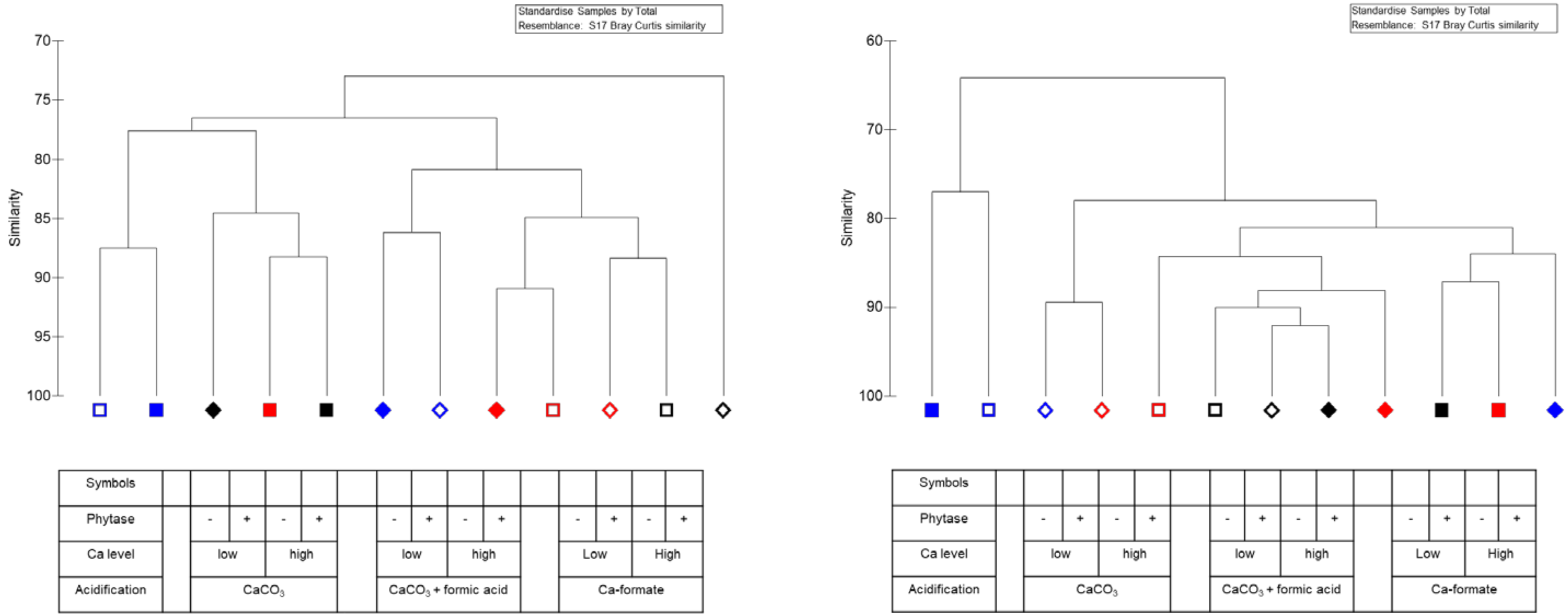

Figure S1. Cluster analysis for the percentage of similarity between the different dietary treatments crop content (left panel) and ileum digesta (right panel).

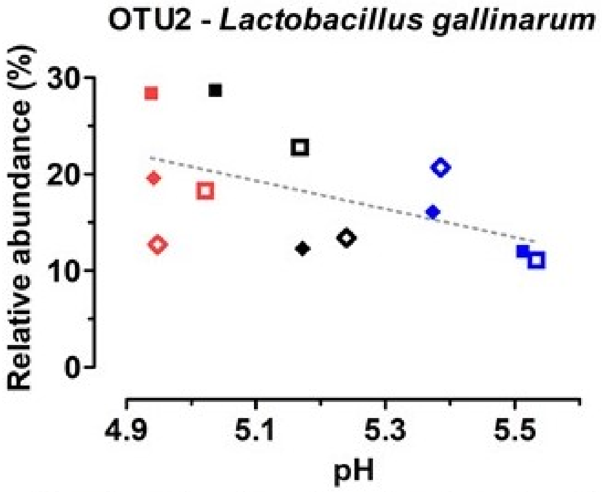

Symbols		□	◇	■	◆		□	◇	■	◆		□	◇	■	◆
Phytase		-	+	-	+		-	+	-	+		-	+	-	+
Ca level		low		high			low		high			Low		High	
Acidification		$CaCO_3$					$CaCO_3$ + formic acid					Ca-formate			

Figure S2. Relationship between crop pH and relative abundance of OTU2 (assigned to *Lactobacillus gallinarum*) in the crop content.

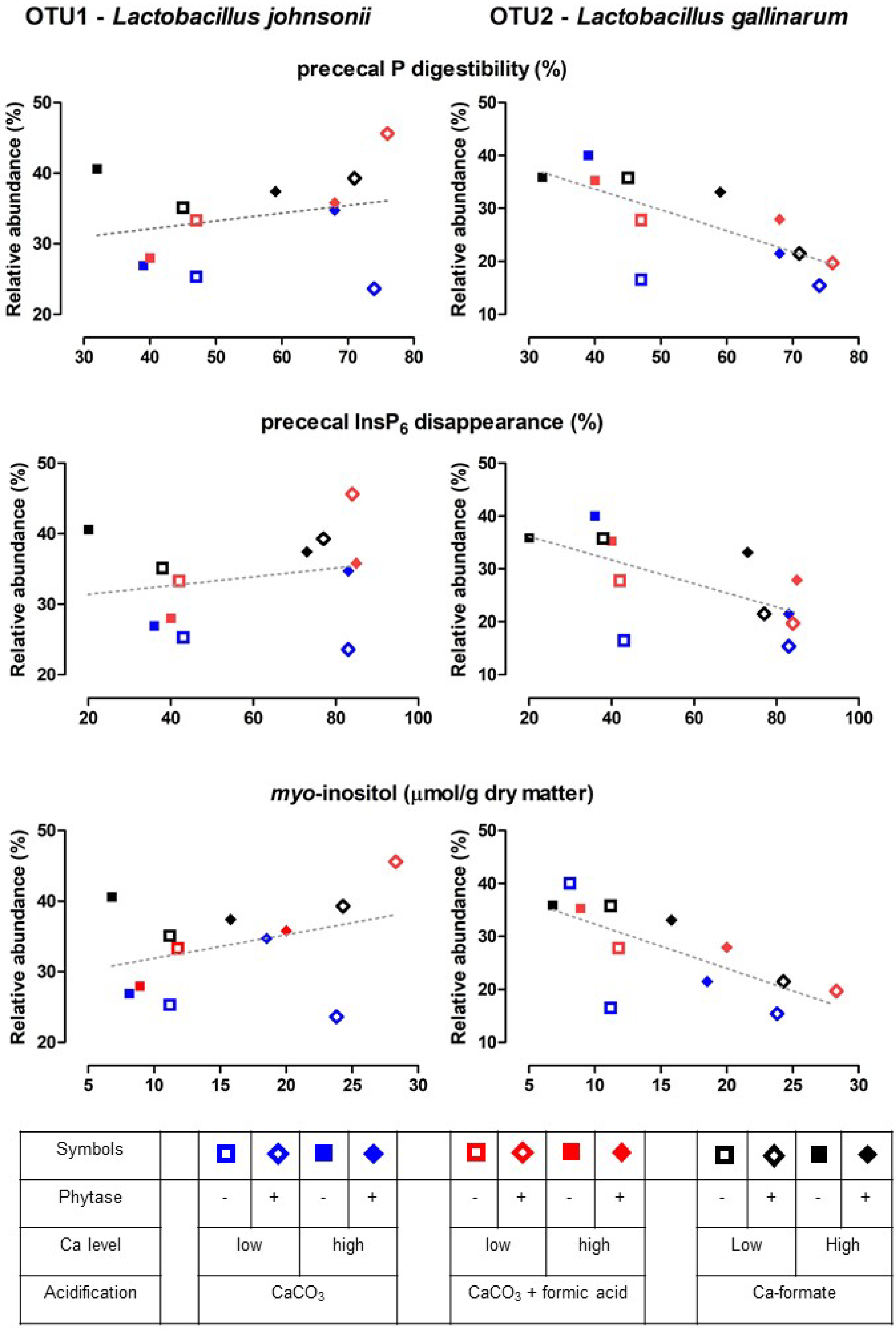

Figure S3. Relationship between relative abundance of OTU1 and OTU2 (assigned to *Lactobacillus johnsonii* and *L. gallinarum*, respectively), being the most abundant OTUs, with prececal P digestibility, prececal $InsP_6$ disappearance, and *myo*-inositol concentration in ileum digesta.
